# Examining the Spin Structure of Altermagnetic Candidate MnTe Grown with Near Ideal Stoichiometry

**DOI:** 10.1021/acsnano.6c01072

**Published:** 2026-07-28

**Authors:** Qihua Zhang, Christopher J. Jensen, Alexander J. Grutter, Sandra Santhosh, William D. Ratcliff, Julie A. Borchers, Thomas W. Heitmann, Narendirakumar Narayanan, Timothy R. Charlton, Mingyu Yu, Ke Wang, Wesley Auker, Saurav Islam, Nitin Samarth, Stephanie Law

**Affiliations:** † Two Dimensional Crystal Consortium Materials Innovation Platform, 8082 The Pennsylvania State University, University Park, Pennsylvania 16802, United States; ‡ NIST Center for Neutron Research, 10833 National Institute of Standards and Technology, Gaithersburg, Maryland 20899, United States; § Department of Physics, The Pennsylvania State University, University Park, Pennsylvania 16802, United States; ∥ Department of Physics, University of Maryland, College Park, Maryland 20742, United States; ⊥ Department of Materials Science and Engineering, University of Maryland, College Park, Maryland 20742, United States; # University of Missouri Research Reactor (MURR), University of Missouri, Columbia, Missouri 65211, United States; ∇ Neutron Scattering Division, 6146 Oak Ridge National Laboratory, Oak Ridge, Tennessee 37831, United States; ○ Department of Materials Science and Engineering, 5972 University of Delaware, 201 Dupont Hall, 127 The Green, Newark, Delaware 19716, United States; ◆ Materials Research Institute, The Pennsylvania State University, University Park, Pennsylvania 16802, United States; ¶ Department of Materials Science and Engineering, The Pennsylvania State University, University Park, Pennsylvania 16802, United States; †† Argonne National Lab, Lemont, Illinois 60439, United States; ‡‡ Institute of Energy and the Environment, The Pennsylvania State University, University Park, Pennsylvania 16802, United States

**Keywords:** molecular beam epitaxy, MnTe, altermagnetism, spintronics, polarized neutron reflectometry

## Abstract

Altermagnets are a recently discovered class of materials with magnetic ordering that have a zero net magnetization and a momentum-dependent spin splitting in their band structure, arising from a collinear spin arrangement with alternating polarizations in the crystal lattice. The nickeline-structured manganese telluride (α-MnTe) is an attractive altermagnet candidate due to its predicted large spin splitting energy and a transition temperature near 300 K. In this work, we present a thorough investigation of the spin structure of α-MnTe thin films grown by molecular beam epitaxy with very high crystal quality and low residual magnetization. The epitaxial α-MnTe films have a full-width-at-half-maximum of 0.1° as measured by X-ray-diffraction rocking curves and a root-mean-square roughness below 1 nm. Neutron diffraction measurements confirm the antiferromagnetic order in the α-MnTe film and show a Néel temperature of 307 K. Polarized neutron reflectometry detects a vanishingly small net magnetization which may be confined to the MnTe/InP interface, which is evidence of the near-ideal stoichiometry in the sample. In vacuo angle resolved photoemission spectroscopy reveals that the bulk band spectrum of the MnTe films is consistent with the weak altermagnetic order as theoretically predicted and observed for the high symmetry nodal plane in the center of the Brillouin zone. This study establishes optimized growth conditions for the synthesis of stoichiometric α-MnTe thin films which exhibit exceptional structural and magnetic ordering, thereby providing a robust platform for evaluating the properties relevant to its consideration as an altermagnetic candidate.

## Graphical abstract

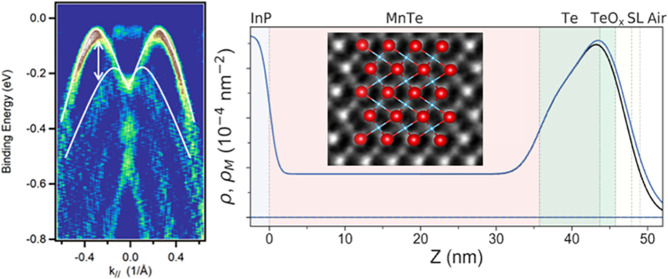

## Introduction

Recently, a new class of magnetic materials known as “altermagnets” has attracted significant research interest since their magnetic properties are unique compared to ferromagnets (FMs) and antiferromagnets (AFMs).^1–10^ Altermagnets feature a net zero magnetization in their bulk form similar to a traditional AFM, and they exhibit a collinear spin arrangement with alternating spin polarizations in their crystal lattice. However, they also possess an anisotropic spin splitting in their electronic band structure, a feature akin to FMs.^11^ The unique properties of altermagnets give rise to a myriad of potential applications in a range of fields including spintronic devices, quantum computing, high-density data storage, and so on. The Nickeline-structured α-MnTe is one of the most attractive altermagnetic materials among all the theorized candidates due to its near room temperature Néel temperature and large predicted spin splitting energy.^12–15^ Historically, α-MnTe was classified as an A-type antiferromagnet with a Néel temperature (*T*_N_) of ∼310 K.^13,16–21^ The spins are ferromagnetically aligned within each basal plane which are oriented antiparallel to each other along the [000*l*] *c*-axis direction. Prior reports confirm that the Néel vector orientation is parallel to the basal plane with negligibly small canting along the [000*l*] direction.^16–18^ It was not until very recently that Šmejkal et al. used *ab initio* calculations to demonstrate that altermagnetic α-MnTe features a *g*-wave anisotropy and a large spin splitting energy of 1.1 eV in the valence band, which is one of the largest predicted among all altermagnetic semiconductor candidates.^2^

With its established altermagnetic candidacy, substantial work has thus been focused on reassessing MnTe to measure its altermagnetic characteristics.^22–32^ A number of reports examining the altermagnetic properties of α-MnTe thin films using angle-resolved photoemission spectroscopy (ARPES), transport measurements, and X-ray absorption spectroscopy have been reported in the past. By combining ARPES data with disordered local moment (DLM) calculations, splitting of the valence band that disappeared as the temperature rose above the Néel temperature was observed, suggesting a connection between the valence band splitting and altermagnetic spin splitting order.^26^ Furthermore, by combining X-ray magnetic linear dichroism (XMLD) and X-ray magnetic circular dichroism (XMCD) with photoelectron emission microscopy (PEEM) measurements, the mapping of altermagnetic order vector was revealed, which showed controlled formations of altermagnetic configurations of diverse sizes, spanning from nanoscale vortices to microscale domains.^23,27^

It is worthy to note that most of these investigations have been performed predominantly on α-MnTe thin films epitaxially grown on InP(111) substrates, owing to the less than 1% lattice mismatch between the *a* lattice constant of MnTe­(4.14–4.15 Å) and the surface lattice constant on (111) plane of InP­(4.15 Å). The electronic band structure of the MnTe/InP system has been studied using density functional theory (DFT) calculations.^18,22,26,33,34^ Nonrelativistic DFT demonstrated a strong altermagnetic spin splitting away from nodal planes purely from exchange interactions and altermagnetic symmetry. When spin–orbit coupling is included, relativistic DFT reveals a weak spin splitting on symmetry-enforced nodal planes that exists in the altermagnetic phase. These theoretical predictions are corroborated by ARPES measurements on MnTe/InP(111) films, which observe weak altermagnetic band splitting at high-symmetry momentum locations such as the Γ–*M*–*K* and Γ–*A*–*H*–*K* nodal planes due to spin–orbit coupling, alongside a much larger nonrelativistic spin splitting away from these planes associated with the strong altermagnetic lifting of Kramers degeneracy.^22^

On the other hand, the detailed magnetic and spin structure of pure α-MnTe thin films to date remains unclear and may be influenced by strain and/or stoichiometry deviations. Other studies, however, indicate that the bulk antiferromagnetic order is preserved in film form.^18,19,35^ Specifically, it is unknown if defect-free films have an intrinsic canted magnetic moment that gives rise to a small net ferromagnetic magnetization. Although Chicote et al. investigated the spin structure and spin asymmetry of α-MnTe film using polarized neutron reflectometry (PNR), the film showed a nonuniform stoichiometric ratio as suggested by a graded nuclear scattering length density (ρ) in the MnTe region as well as a surface oxide layer of 6–7 nm thick.^35^ A significant net ferromagnetic moment (i.e., depth-averaged magnetization of 24 kA/m through the film) was indicated by the spin asymmetry between the reflectivity polarization states, and the magnetic scattering length density (ρ_M_) modeled in the structures highlights the difficulty in classifying the origin of the net magnetization in the MnTe film. Other past reports also acknowledge the existence of substantial net magnetization in the α-MnTe films and attribute it to either a high density of structural defects or intermixing in the film/substrate interfacial region.^18,21,36–39^ A single-phase α-MnTe thin film with careful control of stoichiometric ratio and pristine surface is thus needed to fully understand its spin structure and altermagnetic properties.

Here, we present detailed investigations of the magnetic spin structure of high-quality epitaxial MnTe thin films, with carefully controlled stoichiometry, grown by molecular beam epitaxy (MBE). By varying the growth parameters including substrate temperature and Te/Mn flux ratio (FR), we obtain α-MnTe thin films grown on InP(111) A substrates with high structural quality, as characterized by a surface roughness *R*_q_ ∼ 1 nm (over 2 × 2 μm^2^) and a narrow full-width-at-half-maximum (FWHM) of 0.1° and 0.3° at the MnTe 0004 and 101̅5 X-ray-diffraction (XRD) rocking curves. We use scanning transmission electron microscopy (STEM) and high angle neutron diffraction to confirm the Nickeline crystal structure and the Néel temperature of the α-MnTe film. Our PNR measurements reveal that the α-MnTe film structure is uniform through its depth. In addition, we find an average net magnetization throughout the film of 2.6 kA/m ± 0.5 kA/m, which is more than 2–4 times smaller than previously reported PNR works on α-MnTe films.^19,35^ This improvement in net magnetization likely stems from either a reduced number of defects in the MnTe film or an improved MnTe/InP interfacial region, which denotes a near ideal film stoichiometry. We further use in vacuo ARPES measurements to probe the band structure and find that it corresponds well with previous reports.^22,24^ This paper establishes growth conditions and protocols that allow the synthesis of stoichiometric α-MnTe films of high structural and magnetic order, thus providing a platform for careful evaluation of properties relevant to altermganetism that are not influenced by extrinsic factors such as inhomogeneous ferromagnet phases.

## Results and Discussion

### Optimization of Growth Conditions for α-MnTe Layers

#### Growth Temperature

We first investigated the effect of growth temperature over a range of 200–500 °C (measured by thermocouple) on the quality of MnTe films. The samples in this series were grown with a fixed growth rate of 0.35 Å/s (1 Å = 0.1 nm), a fixed Te/Mn flux ratio (FR) of 3, and a constant growth duration of 20 min. All samples have similar MnTe film thickness of 26–29 nm as measured by X-ray reflectivity (XRR) fitting, suggesting that the desorption rate for Mn adatoms is minimal within this wide growth temperature window. We first compared the surface morphology of Samples A–F, grown with a temperature of 200 °C (Sample A), 250 °C (Sample B), 300 °C (Sample C), 340 °C (Sample D), 400 °C (Sample E), and 500 °C (Sample F), respectively. As seen in Figure 1, triangular-like and hexagonal-like grains on all the six samples are observed, consistent with the desired hexagonal-phase MnTe. However, Sample A, grown with a temperature of 200 °C, has a number of needle-like whiskers on the surface. We suspect that these whiskers are MnTe_2_ pyrite crystals formed randomly on the surface, owing to the low diffusion length of both Mn and Te adatoms from the low growth temperature; however, the quantity of the whiskers is insufficient to be detected by XRD scans. By increasing the growth temperature from 250 to 500 °C, one can see clear step edge formation along with increasing grain sizes due to the increase in adatom mobility and a disappearance of the whiskers. However, the surface also becomes more columnar and island-like, with RMS roughness gradually increasing from ∼1.0 to 3.0 nm from Sample B to F. This suggests that a high growth temperature is not favorable for MnTe growth, as the high desorption rate of the Te adatoms compromises the MnTe nuclei formation during the initial stages of growth.

**Figure 1:**
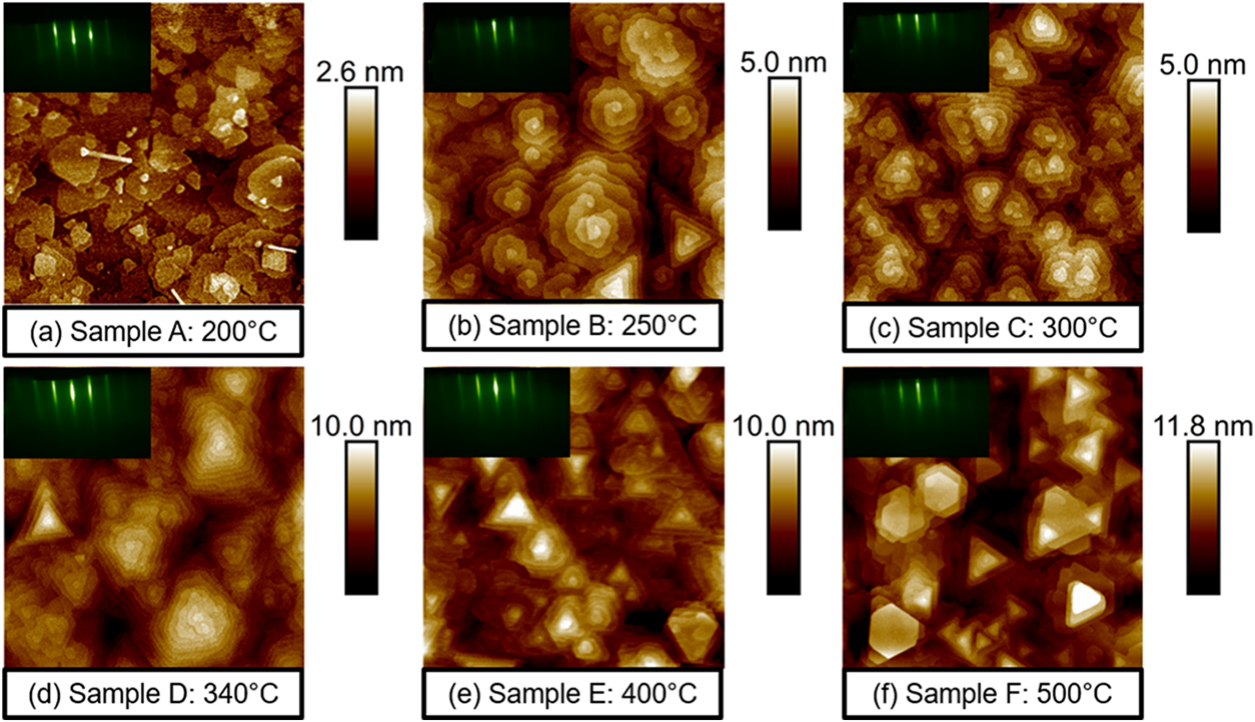
AFM images of MnTe films grown with growth temperatures of (a) 200 °C, (b) 250 °C, (c) 300 °C, (d) 340 °C, (e) 400 °C, and (f) 500 °C. All AFM scans are 2 × 2 μm^2^. The inset displays the corresponding RHEED images captured immediately after growth at the growth temperature.

As both Mn and Te fluxes were kept constant in this series of samples, the increased Te desorption rate with the increased growth temperature reduces the effective Te/Mn ratio at the growth front, which in turn reduces the crystalline quality of the synthesized MnTe films. To confirm this, we carried out high resolution XRD studies of Samples A–F. Exemplary XRD scans including the 2θ-ω and the ω-scans (i.e., rocking curves) of the MnTe 0004 and 101̅5 reflections are shown in Figure S1. Using the MnTe (0004) 2θ peak position at ∼54.71°, the *c*-axis lattice constant of MnTe film in Sample A is calculated to be ∼6.71 Å, which agrees very well with the bulk MnTe values reported in past studies.^12,13,18^ We note that the FWHM values in the XRD rocking curves for both the (0004) and (101̅5) reflections (0.09° and 0.21°, respectively) as presented in Figure S1­(b,c) are among the lowest for heteroepitaxial chalcogenide thin films that are only ∼30 nm thick; this high quality is expected due to the small lattice mismatch between MnTe and InP.^40^

Figure S2­(a) plots the FWHM values of the XRD rocking curves for the MnTe 0004 symmetric reflection and the 101̅5 asymmetric reflection as a function of growth temperature. We see that the FWHM values in both MnTe reflections increase with the increasing growth temperature, indicating an increase in threading dislocations in the film. This confirms that the high Te desorption rate at the high growth temperature degrades the crystalline quality. In summary, we find that a lower growth temperature improves the crystal quality of MnTe layers but promotes the growth of surface features such as whiskers. We therefore conclude that the optimized window for the growth temperature is 250–400 °C.

#### Te/Mn Flux Ratio

Based on the results from growth temperature study, we determined that controlling the Te desorption is critical to improving the MnTe film quality as it heavily influences the dynamics at the growth front. Therefore, we further studied the effect of Te/Mn FR on the MnTe film quality. In this series, the growth temperature and growth rate were fixed at 340 °C and 0.35 Å/s, respectively. Figure 2a–d displays the AFM images of MnTe layers grown with a Te/Mn FR of 1 (Sample G), 2 (Sample H), 3 (Sample D), and 4 (Sample J). Although the RMS roughness in Sample G (Te/Mn = 1) is the lowest among the four at 0.8 nm, the morphology in this sample is granular-like and noncontinuous. The small, dense grains are rounded and globular, which is not consistent with the hexagonal- or triangular-like grains that are typically observed in (0001)-oriented thin films. We speculate that these grains are Mn droplets or Mn clusters, which are caused by the insufficient Te supply at the growth front. As the Te/Mn FR increases from 2 to 4 (Figure 2b–d), the RMS roughness decreases from 2.0 to 1.2 nm. This effect can be understood by realizing that the increasing Te supply compensates for the high Te desorption rate at the growth temperature. This is also consistent with the increased surface roughness with increasing growth temperature as discussed earlier. We therefore determine that a high Te/Mn FR benefits the surface morphology of MnTe films. Unfortunately, in this study, the maximum Te flux we can achieve is ∼3.1 × 10^14^ cm^–2^ s^–1^, which is very close to the physical and thermal limitation of the Te effusion cell. We also note that maintaining a high operating temperature for Te effusion cell increases the likelihood of clogging in the effusion cell.

**Figure 2:**
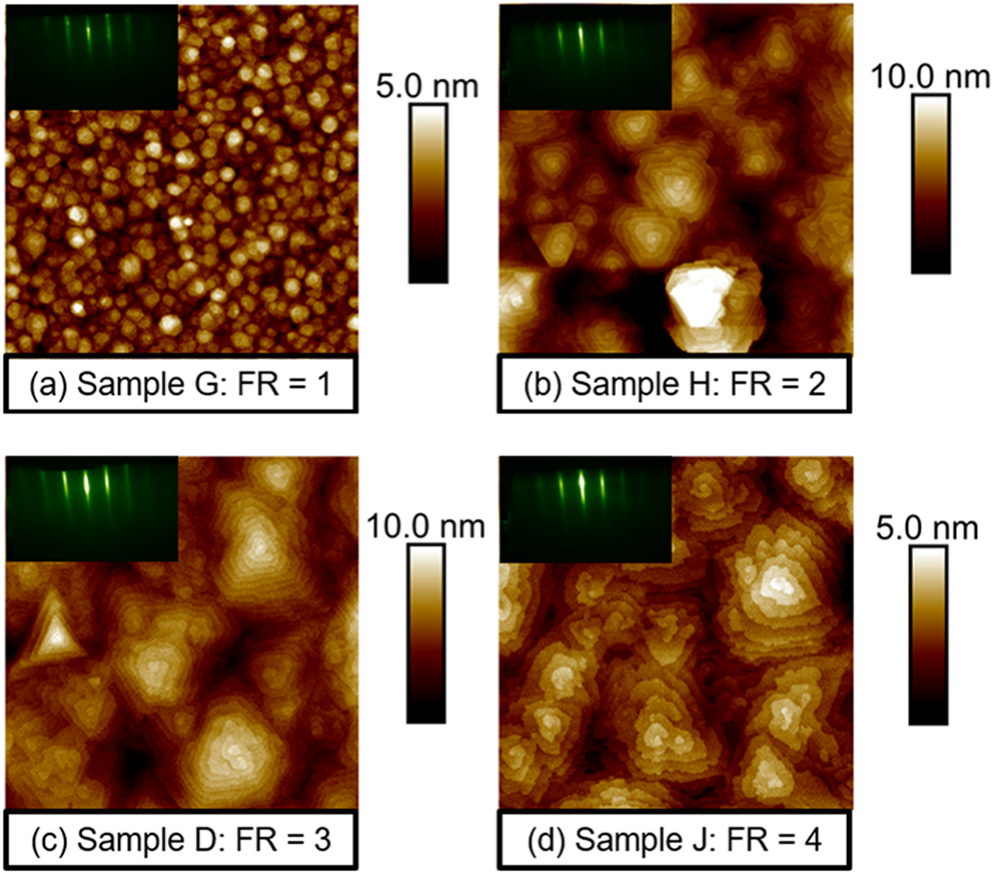
AFM images of MnTe films grown with Te/Mn flux ratios of (a) 1, (b) 2, (c) 3, and (d) 4. Sample D is repeated from Figure 1 and displayed again for comparison purposes. All AFM scans are 2 × 2 μm^2^. The RMS roughness in the images is (a) 0.8 nm, (b) 2.0 nm, (c) 1.6 nm, and (d) 1.2 nm. The inset displays the corresponding RHEED images captured immediately after growth at the growth temperature.

In addition to the AFM scans, we can also compare the crystalline and structural quality of the MnTe films in this series with XRD. The FWHM values of the MnTe (0004) and (101̅5) reflections XRD rocking curves are plotted as a function of Te/Mn flux ratio in Figure S2­(b). The FWHM of the MnTe (0004) rocking curves for all four samples are in the range 0.1°–0.2°. It is again clear that a higher Te/Mn FR is beneficial to the crystalline quality of the MnTe layers, since the FWHM values of the MnTe (0004) and (101̅5) rocking curves both decrease with the increasing Te flux. On the other hand, oversupplying Te flux will eventually undermine the film quality either due to a decrease in the Mn adatom surface diffusion length causing cluster formations or due to the formation of additional phases such as MnTe_2_.

### Structural Characterization of Epitaxial MnTe Films

Taking into consideration the limits of the Te effusion cell, we find an optimal growth window for growth temperature and Te/Mn flux ratio to be ∼250 °C–350 °C and 3, respectively, where the MnTe films have a clean surface as well as a narrow FWHM in XRD rocking curves. We have analyzed the structural characteristics of MnTe films grown in this window. Figure 3a shows reciprocal space mapping (RSM) of Sample D around the asymmetric InP(224) diffraction. A mosaic of MnTe(101̅5) diffraction is also captured in addition to the anticipated InP(224) diffraction, where the in-plane component (*Q*_*x*_) of MnTe matches closely that of InP. Using the center of the mosaic pattern, the in-plane lattice constant *a* of MnTe is calculated to be 4.17 Å, which is slightly larger (<0.8%) than the value reported in bulk crystals (4.15 Å),^43^ and indicates that such 30 nm MnTe layer is nearly fully relaxed with minimal residual strain.

**Figure 3:**
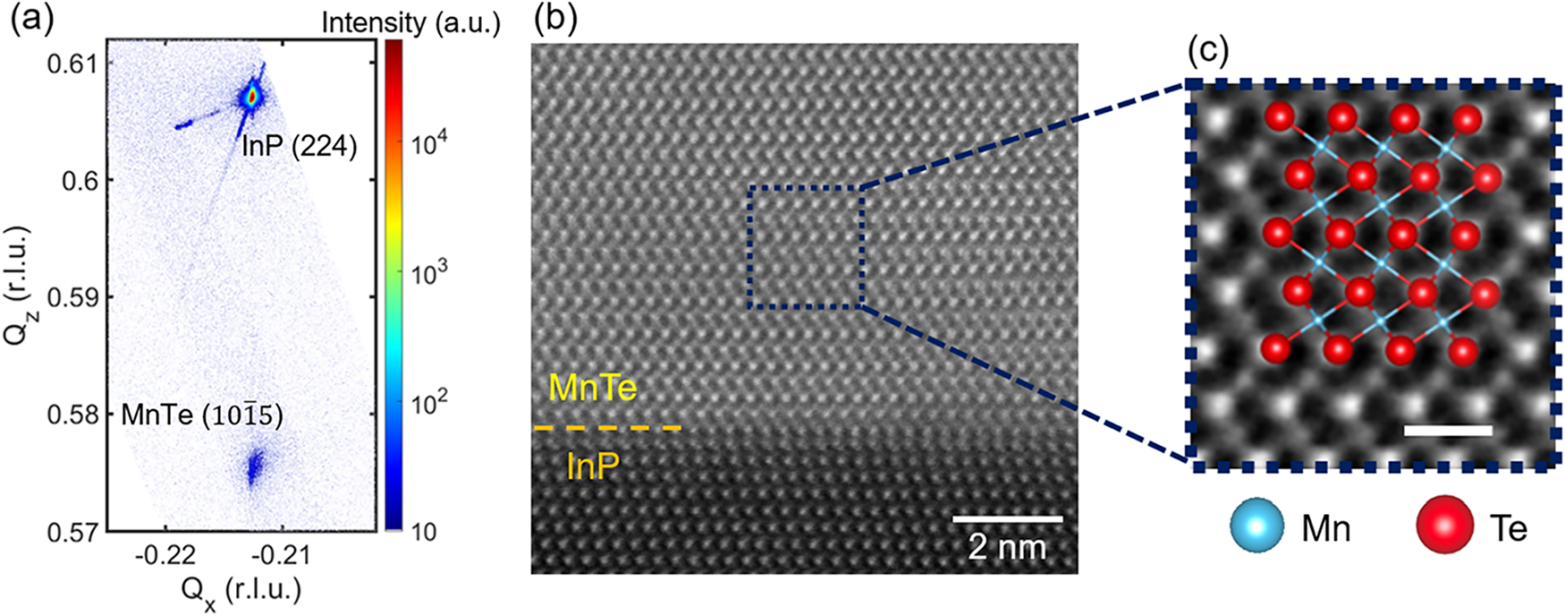
(a) XRD reciprocal space maps around the InP (224) diffractions. Both InP(224) and MnTe(101̅5) mosaics are labeled. (b) HAADF-STEM image showing the MnTe/InP interface. The yellow dashed line indicates the location of MnTe/InP interface. (c) High magnification HAADF-STEM image of the boxed region in (b), superimposed with the NiAs-type crystal structure created in VESTA. The scale bar in (c) is 0.5 nm.

Cross-sectional TEM studies were carried out to characterize the structural properties of the MnTe films grown with conditions identical to Sample D but with a film thickness of ∼100 nm. A low-magnification cross-sectional HAADF-SEM image along with the In, Mn, and Te energy dispersive spectroscopy mapping is shown in Figure S3. The sharp and clear transition from the In signal to the Te and Mn signals suggests an abrupt interface between the substrate and the epitaxial film. We also note that a thin amorphous oxide layer has formed on top of the sample, which is likely due to the natural oxidation of the film. High-resolution HAADF-STEM images are shown in Figure 3b,c, further confirming the sharp interface between MnTe and InP. We see that the as-grown MnTe film is the α-polytype by superimposing the NiAs-type hexagonal crystal lattice on top of the HAADF-STEM image in Figure 3c.^44^ It is also worth noting that upon inspecting the entire FIB sample, no additional polytype or phases of MnTe are found, consistent with the XRD scans and again confirming the polytype purity and excellent quality of the MnTe film.

### Neutron Diffraction

High angle neutron diffraction measurements were performed on 35 and 100 nm MnTe films which were grown with identical conditions to Sample D to determine if the magnetic structures of these films are consistent with bulk behavior. A distinct (0001) peak, which is structurally forbidden, appears at low temperature, confirming that antiferromagnetic order comparable to bulk develops in both films. The temperature dependence of the integrated intensity of the antiferromagnetic peak is plotted in Figure 4a,b for the 35 and 100 nm films, respectively, and follows the expected order parameter behavior. Fits of these data to a Brillouin function indicate that *T*_N_ = 307 ± 2 K for the 35 nm film and 304 ± 1 K for the 100 nm film. These values are approximately equal to the bulk ordering temperature and indicate that the antiferromagnetic component film magnetism mimics that of bulk MnTe.

**Figure 4:**
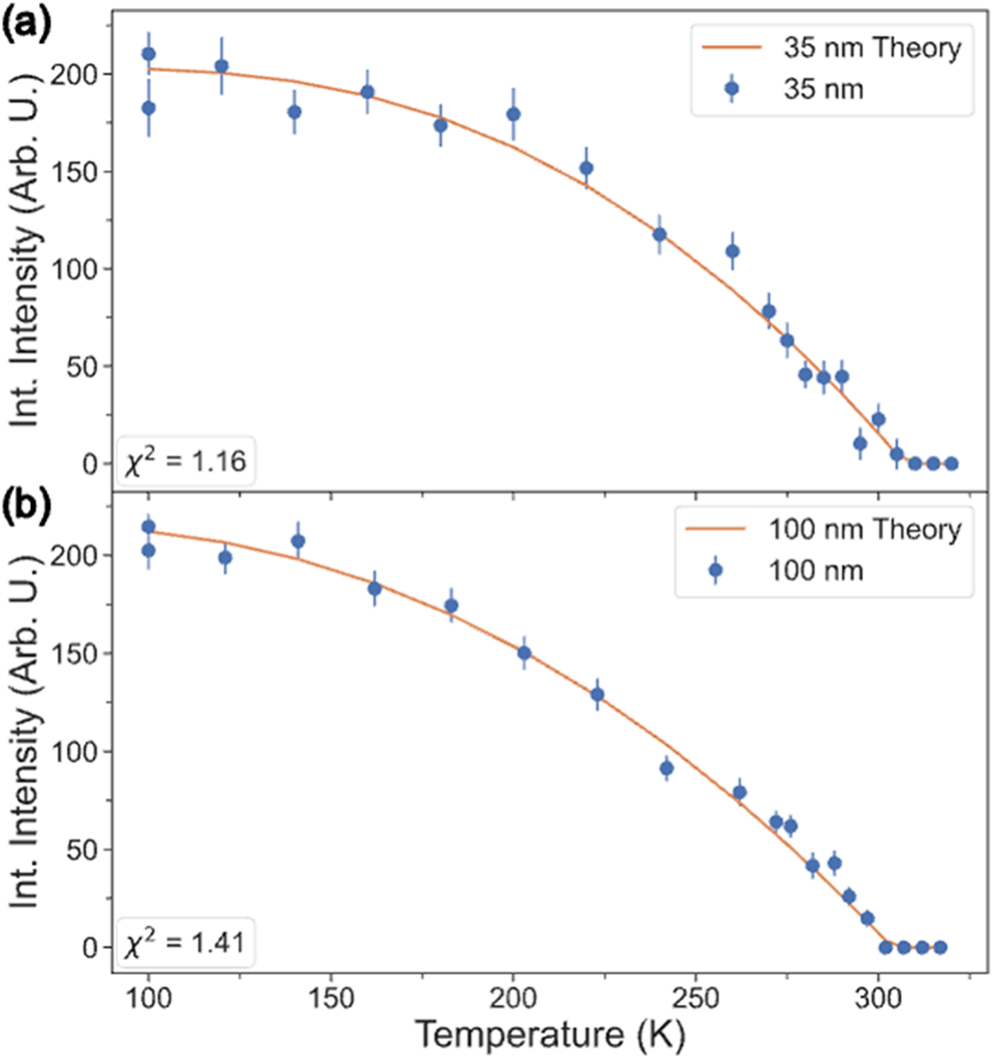
Integrated intensity of the (0001) antiferromagnetic reflection as a function of temperature for (a) a 35 nm MnTe film and (b) a 100 nm MnTe film. The lines correspond to a fit to a Brillouin function.

### Polarized Neutron Reflectometry

To explore the possibility of an additional ferromagnetic component of the MnTe magnetization, PNR measurements were taken at 20 and 100 K, below the *T*_N_ of MnTe, in a 1 T magnetic field directed within the film’s surface. This field was expected to align any ferromagnetic (FM) components of the MnTe magnetization into the plane of the film along the field direction. In this experiment, the 35 nm MnTe film is again grown with growth conditions identical to Sample D then capped with a 5 nm amorphous Te layer. Two models of the scattering length density profiles as a function of depth (Figure 5a,c) generated best fits of equivalent quality, and so we could not rule out either as a potential solution. In the first model, referred to as the “bulk” model, the magnetization ρ_M_ in the MnTe layer was uniform. Figure 5a shows the resulting ρ and ρ_M_ profiles for the two measurement temperatures, with the ρ_M_ profile rescaled in Figure 5b for clarity. Fit independently, the other model, referred to as the “interfacial magnetization” model, converged to a profile with all the same characteristics of the bulk model (within error), except ρ_M_ in MnTe was decoupled from the thickness of the layer (Figure 5c,d). A feature of both models is that the fitted nuclear ρ is uniform through the entire MnTe layer thickness, and the values obtained in these fits are 0.440 × 10^–4^ nm^–2^ (bulk model) and 0.446 × 10^–4^ nm^–2^ (interfacial magnetization model). The 95% confidence intervals (CI) for these models ρ are 0.421–0.457 × 10^–4^ nm^–2^ and 0.428–0.461 × 10^–6^ nm^–2^, for the bulk and interfacial magnetization models, respectively, which are slightly higher than the bulk ρ value of MnTe of 0.379 × 10^–4^ nm^–2^. This result suggests that the structural quality of the film is comparable to that of bulk crystals, though slight Mn-deficiency or strain compression along the *c*-axis may give rise to a slight increase in the apparent density. The latter, however, is unlikely since the lattice parameters measured by XRD approximate those of bulk.

**Figure 5:**
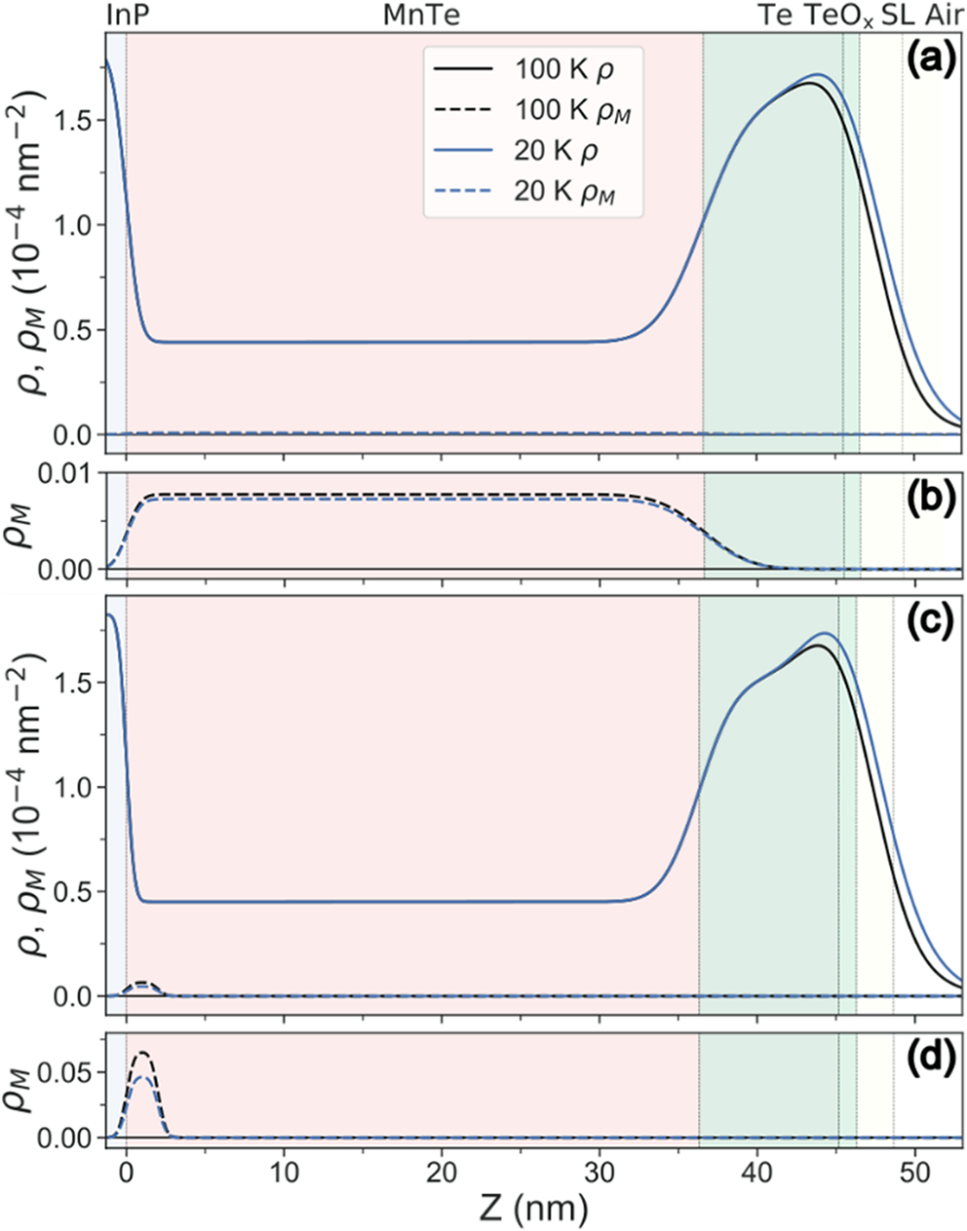
PNR ρ and ρ_M_ profiles calculated from reflectivity fits of Te/MnTe at 100 K (black) and 20 K (blue) from the bulk layer model (a, b) and the interface magnetism model (c, d). Solid lines represent ρ and the dashed lines represent ρ_M_ (a, c) and zoomed regions of ρ_M_ (b, d) were used to illustrate the magnetic profiles. Background colors with corresponding labels above the plot highlight expected layer positions along the sample depth (Z). “SL” represents a surface layer of ice/condensation formed during sample cooling.

The depth profiles in Figure 5 were generated from the reflectivity data and fits for the 20 K measurements shown in Figure 6a (bulk model) and Figure 6c (interfacial magnetization model). It should be noted that the discontinuity in the data results from the different detector angles used during the time-of-flight measurements, with overlapping regions in the data along Q. The 100 K reflectivity data (Figures S4–S7 in Supporting Information) did not vary significantly from the 20 K data. The reflectivity fits corresponding to both models, shown as solid lines, are in good agreement with the data, and have similar goodness of fit (χ^2^), highlighting their equivalence as potential solutions.

**Figure 6:**
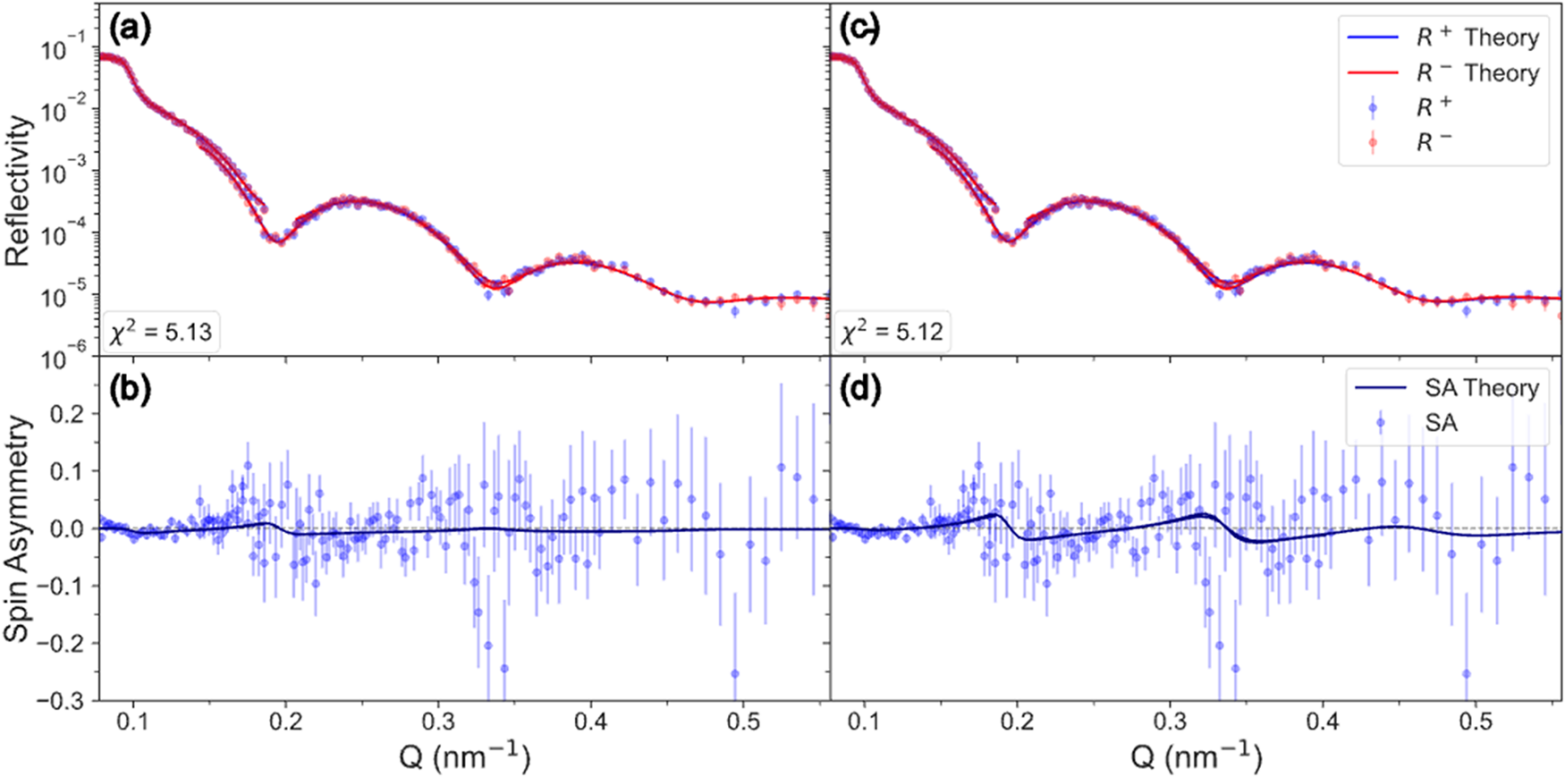
PNR data (points) and theoretical curves (solid lines) calculated from fits using the bulk layer (a, b) and interfacial magnetization models (c, d) for measurements at 20 K on Te (10 nm)/MnTe (35 nm)/ InP films. The reflectivity data and fits (a, c) were used to calculate the spin asymmetry (b, d) defined as (R^+^ – R^–^)/(R^+^ + R^–^). Error bars on the reflectivity data and calculated spin asymmetry represent the standard deviation.

Spin asymmetry—defined as (R^+^ – R^–^)/(R^+^ + R^–^)—is useful for highlighting the differences between the two polarization states that arise from the presence of in-plane magnetization. The spin asymmetry data and the corresponding fits for the bulk model (Figure 5a,b) and interfacial magnetization model (Figure 5c,d) are shown in Figure 6b,d, respectively. In general, the magnitude of the spin asymmetry is small, and the variations about zero are mostly within the level of uncertainty—indicative of a small magnetic contribution in the sample. While, qualitatively, the interfacial magnetization model seems to better capture the shape of the spin asymmetry, the large uncertainty above *Q* ≈ 0.25 nm^–1^ limits the determination of the true shape of the data—again suggesting equivalence in the models as potential solutions.

Table 1 summarizes the 100 and 20 K fit values for ρ_M_ for the bulk and interfacial magnetization models and their 95% confidence interval (CI), calculated from uncertainty analysis with a Markov Chain Monte Carlo method (Bayesian approach).^45^ The 95% CI does not contain zero for either model, suggesting a finite magnetization persists in both cases. For the bulk model (Figure 5b), a uniform, vanishingly small ρ_M_ resulted from fits of both the 100 and 20 K data. In the interfacial magnetization model (Figure 5d), ρ_M_ is significantly larger, but is confined to a thickness of 1.38 nm ± 0.87 nm at the MnTe/InP interface (for both 100 and 20 K). We find no evidence for a transitional interface region, as models which allowed for an interface region with an ρ distinct from that of the rest of the MnTe film did not improve the fit (Figures S10 and S11 in Supporting Information). PNR is therefore consistent with a uniform structure throughout the entire film depth.

**Table 1:** ρ_M_ Fit Values and Uncertainty for the Bulk and Interfacial Magnetization Models

model	*T* (K)	ρ_M_ (×10^‑4^ nm^–2^)	*M*_fit_ (kA/m)	95% CI (×10^‑4^ nm^–2^)	*M*_max_ (kA/m)
Bulk	100	0.0080	2.8	0.0033–0.0123	4.31
	20	0.0074	2.6	0.0042–0.0105	3.68
Interfacial Mag.	100	0.134	48	0.03–0.36	130
	20	0.094	33	0.02–0.25	88

To explore the most extreme case, a maximum, laterally averaged magnetization, *M*_max_, was calculated from the upper 95% CI bound using the conversion constant *c* = 2.853 × 10^–7^ nm^–2^ kA^–1^ m (Table 1). For the bulk model, the *M*_max_ ≈ 4 kA/m is effectively negligible, but would be consistent with a very small number of FM impurities and/or clusters uniformly distributed throughout the bulk of the MnTe layer. For the interfacial magnetization model, it should be noted that the majority of the film has zero magnetization, and the values indicated in the table represent only the thin sublayer at the MnTe/InP interface with *M*_max_ ≈ 90–130 kA/m. Such a localized magnetization could be caused by a small amount of diffusion and/or strain isolated in the thin interfacial layer, though the uniformity of the nuclear ρ profile (Figure 5c) does not support this interpretation. Similarly, a slight increase in Mn content has been attributed to FM in MnTe,^35^ but this does not explain FM in either of our films since we observed a uniform increase in ρ in the MnTe layer from the expected value, consistent with a Mn deficiency.

While the above analysis focuses on the upper limit of the MnTe magnetization established by PNR, the best-fit values, M_fit_, for both the bulk and interfacial magnetization models (Table 1) are approximately a factor of 2 more than *M*_max_. In addition, the average magnetization M_fit_ for the bulk model is substantially less than the magnetization measured by neutron reflectivity on previously reported MnTe films,^19,35^ which ranges from 10–50 kA/m. Any ferromagnetic component of the magnetization through the MnTe film is either trace or confined to a very narrow interfacial region. Both possibilities indicate that the high quality MnTe film is bulk-like in character with good stoichiometry and very limited magnetic clustering or local defects.

### Angle Resolved Photoemission Spectroscopy

Theoretical calculations and experimental ARPES measurements on MnTe bulk crystals and thin films have shown altermagnetic lifting of Kramer’s spin degeneracy of bulk bands.^22^ To compare the band structure of our MnTe thin films with prior results, we carried out in vacuo angle resolved photoemission spectroscopy ARPES measurements of 35 nm thick MnTe thin films using a Helium lamp with 21.2 eV photon energy. Prior ARPES experiments on MBE grown MnTe thin films with similar photon energy show that the *k*_*z*_ value is in the high symmetry nodal plane Γ–*M*–*K*.^22^ Below the Néel temperature (307 K, as shown by neutron diffraction), we expect to observe a weak altermagnetic band splitting at the top of the valence band. Figure 7a–c show the constant energy maps of the thin film integrated over 50 meV around the corresponding binding energy value. The low spectral intensity at the Fermi surface, in Figure 7a, indicates the absence of bands crossing the Fermi level at 77 K as expected from the semiconducting nature of our MnTe thin films. From the hexagonal symmetry of the map in Figure 7b,c, we conclude that the sample has equal populations of domains with Néel vector easy axis pointing along three equivalent crystallographic directions (Γ–*M* directions). We also map the band spectrum (momentum dispersion curves) along the Γ–*K* (Figure 7d) and the Γ–*M* (Figure 7f) directions at 77 K. The corresponding second derivatives, Figure 7e,g shows a clear band spectrum resembling the valence band of MnTe, indicating that the film exhibits p-type carrier characteristics. This is consistent with our transport study where a free hole concentration of ∼3 × 10^19^ cm^–3^ was measured (See Figure S12 in Supporting Information). The high spectral intensity bands observed in the measurements are similar to those seen in prior studies^22^ of MnTe films at *k*_*z*_ = 0. A relativistic weak altermagnetic band splitting of magnitude ∼100 meV (Figure 7d,e) is clearly observed along the Γ–*K* direction. Using the second derivative plot, we could also resolve the lower magnitude relativistic splitting <50 meV along the Γ–*M* direction (Figure 7g). White lines are drawn on the second derivative plots along with arrows to emphasize the split band and their magnitude. Our results are consistent with the theoretical predictions and experimental literature on ARPES measurements on MnTe thin films grown on InP(111) substrates.^26^ We note that to confirm the much larger “strong” altermagnetic band splitting, we would need to carry out future ARPES measurements under appropriate conditions that access momentum space away from the nodal planes. We further emphasize that, while our results are consistent with altermagnetic band structure predictions, direct confirmation of altermagnetism would require spin-resolved ARPES measurements resolving the spin splitting, which will be addressed in future investigations.

**Figure 7:**
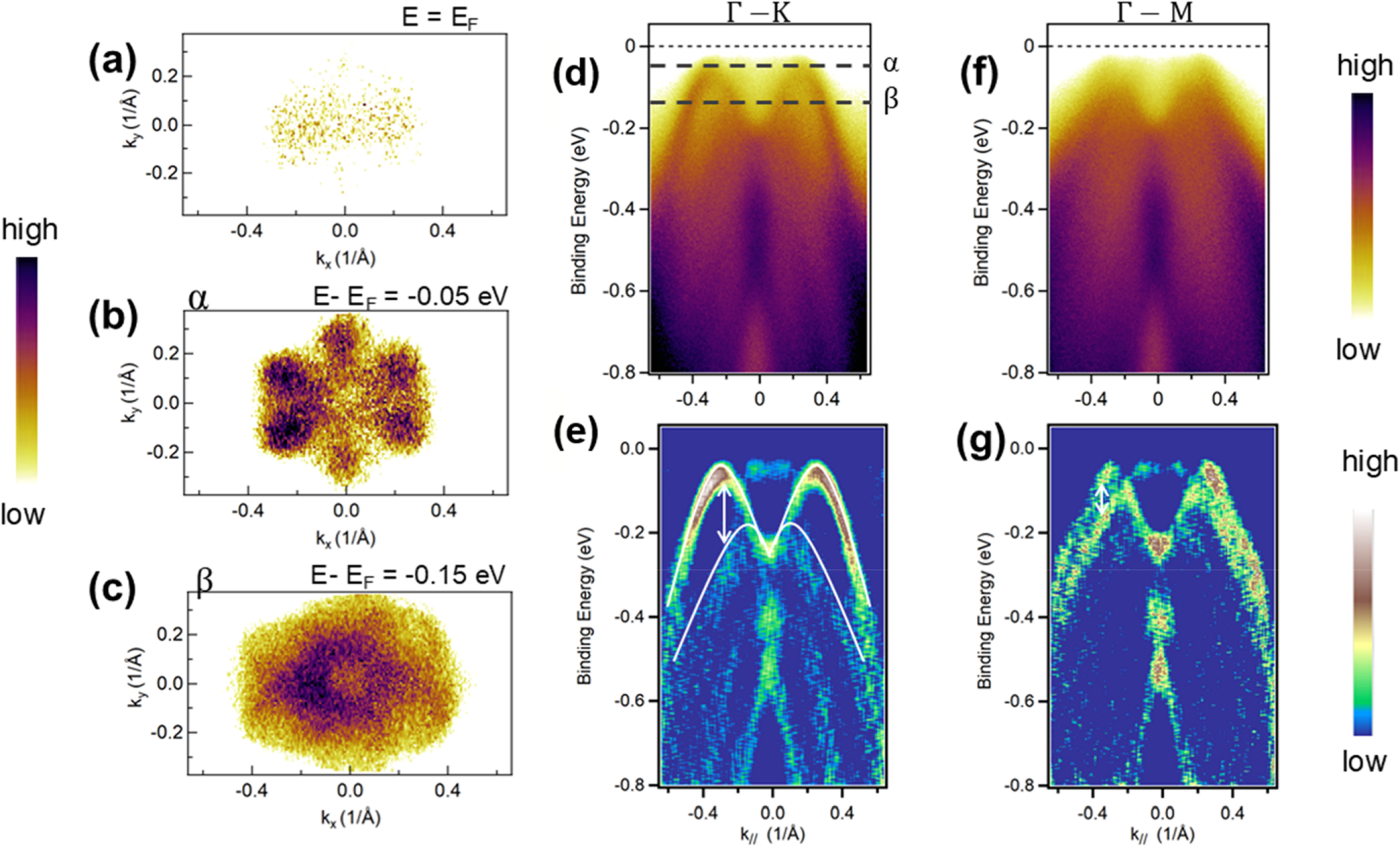
ARPES measurements on 35 nm thick MnTe film: (a–c) Constant energy maps of the film integrated over 50 meV around the binding energy (a) *E* – *E*_F_ = 0 (b) *E* – *E*_F_ = −0.05 eV and (c) *E* – *E*_F_ = −0.15 eV. (d–g) ARPES band spectra measured along the Γ–*K* (d) and Γ–*M* (f) direction. To emphasize the splitting in the valence band, we plot the second-derivative curvature plots of the band spectra along the (e) Γ–*K* and (g) Γ–*M* directions. All ARPES data are measured at 77 K. The white lines and the arrow in (e) and (g) are guides to the eye, indicating the split bands and the magnitude of the splitting, respectively.

## Conclusions

In conclusion, we have reported an in-depth study on the magnetic spin structure of α-MnTe films with exceptional quality. We first identified an optimized growth window of MnTe (film growth rate of 0.35Å/s, Te/Mn flux ratio of 3–4, substrate temperature of 250–400 °C) on InP substrates, which produces ultrahigh quality MnTe films including smooth surfaces with RMS roughness of ∼1 nm and narrow FWHM of 0.1° and 0.3° at MnTe 0004 and 101̅5 XRD rocking curves. A single-phase α-MnTe film was further confirmed with reciprocal space mapping and scanning transmission electron microscopy experiments. Using a 35 nm MnTe film grown with optimized conditions, neutron diffraction was conducted to confirm the antiferromagnet nature of the synthesized film, which has a Néel temperature of ∼307 K. We further used polarized neutron reflectometry to investigate the possibility of a FM component in the MnTe magnetization, with the modeled ρ_M_ indicating a vanishingly small average magnetization of 2.6 kA/m that is more than a factor of 2 to 4 smaller than previously reported values. The small net magnetization is a strong indicator of the film’s high quality and is either attributable to a very small number of impurities spread through the MnTe film or confined to a narrow MnTe/InP interfacial region. Finally, low temperature (77 K) He lamp ARPES measurements reveal the relativistic weak altermagnetic band splitting in 35 nm thick MnTe thin films. These results thus highlight the ultrahigh quality in the synthesized MnTe film with a nearly ideal stoichiometric order.

## Methods and Experimental Section

### MBE Growth

In this report, all MnTe films were grown in a DCA Instruments R450 MBE system (instrument details at DOI: 10.60551/gqq8-yj90) with a base pressure of 5 × 10^–10^ Torr. InP(111)­A wafers diced into 1 × 1 cm^2^ pieces were used as substrates. Standard solvent cleaning procedures (acetone and isopropyl alcohol ultrasonic bath) and thermal outgassing at 200 °C for 2 h in the load lock chamber were performed on the substrates prior to sample growth. After outgassing in the load lock, the substrate was heated to 650 °C at a ramp rate of 30 °C/min in the MBE system and annealed for 10 min with a constant supply of tellurium at a flux of 2.3 × 10^14^ cm^–2^ s^–1^ to desorb the surface oxide.^40^ The substrate temperature was measured by a noncontact thermocouple mounted behind the substrate. A STAIB reflection high electron diffraction (RHEED) electron gun and kSA 400 analytical RHEED software were used to monitor the surface in situ during the substrate anneal and sample growth. Te and Mn fluxes of >5N purity were supplied in standard Knudsen cells. A ColnaTec quartz crystal microbalance (QCM) operating at 6 MHz was used for flux calibrations at the growth position. For the growth optimization studies, a consistent Mn flux of 7.6 × 10^13^ cm^–2^ s^–1^ was used, and growth durations were fixed at 20 min with a target film thickness of ∼30 nm. The Te flux was varied from 7.6 × 10^13^ cm^–2^ s^–1^ to 3.1 × 10^14^ cm^–2^ s^–1^, while the growth temperature varied from 200 to 500 °C. After the film deposition was completed, the sample was annealed for 2 min at the growth temperature with the Te shutter open and then ramped down to room temperature at a rate of 50 °C/min. For samples used for the PNR experiment, a 5 nm amorphous Te layer was deposited on the MnTe sample in the MBE system at room temperature with a Te flux of 1 × 10^14^ cm^–2^ s^–1^ to prevent oxidation of the MnTe layer.

### Angle Resolved Photoemission Spectroscopy (ARPES)

ARPES measurements were performed on a 35 nm thick MnTe film using a Helium lamp with a photon energy of 21.2 eV. The detection of the photoemitted electrons were done using a Scienta Omicron DA 30L analyzer with an energy resolution of 6 meV. The samples were transferred from the growth chamber to an ultrahigh vacuum ARPES chamber using a vacuum suitcase with a base pressure less than 5 × 10^–8^ Torr. The samples were measured at 77 K.

### Structural and Surface Analysis of MnTe Films

High resolution XRD measurements were conducted using a Malvern PANalytical 4-circle X’Pert^3^ system equipped with a hybrid 2-bounce asymmetric Ge(220) monochromator and a PIXcel 3D detector. The surface morphology was examined in a Bruker Dimension Icon atomic force microscope (AFM) with a lateral resolution of 512 pixels/line. A SCANASYST-AIR probe was used for AFM imaging. Thin cross-sectional transmission electron microscope (TEM) specimens were prepared by using focused ion beam (FIB, FEI Helios 660) lift-out technique. A thick protective amorphous carbon layer was deposited over the region of interest prior to FIB, and a 2 kV final cleaning was used to remove the ion beam damage the sample surface. Then the cross-sectional microstructures of the films were observed by FEI Titan3 G2 double aberration-corrected microscope at 300 kV. All scanning transmission electron microscope (STEM) images were collected by using a high-angle annular dark field (HAADF) detector which had a collection angle of 52–253 mrad. EDS elemental maps of the sample were collected by using a SuperX EDS system under STEM mode.

### High Angle Neutron Diffraction

Elastic neutron scattering measurements on 35 and 100 nm MnTe films on InP(111) substrates (lateral area of 1 cm^2^) were performed in zero applied magnetic field at the University of Missouri Research Reactor (MURR) using the triple-axis spectrometer TRIAX. A pyrolytic graphite (PG) monochromator was used to select an incident beam energy of 14.7 meV, and a PG analyzer after the sample was also set to 14.7 meV to reduce the background. Higher wavelength harmonics were reduced with PG filters positioned in the incident and scattered beams. The beam divergence was defined using 60′-60′-80′-80′ collimators positioned before the monochromator, between the monochromator and sample, between the sample and analyzer, and between the analyzer and detector, respectively. The sample was sealed in a ^4^He environment within an aluminum can and subsequently mounted on the cold tip of an Advanced Research Systems closed-cycle refrigerator. The films were aligned with the [000*l*] MnTe growth axis parallel to the wavevector Q direction, and θ/2θ scans were performed through the (0001) antiferromagnetic reflection at different temperatures upon heating the sample. These data were fit with Gaussians, and the integrated peak intensities were plotted as a function of temperature to produce antiferromagnetic order parameters.

### Polarized Neutron Reflectometry

PNR experiments were conducted on the MAGREF reflectometer at the Spallation Neutron Source at Oak Ridge National Laboratory on Te (5 nm)/MnTe (35 nm)/InP films with a lateral area of 1 cm^2^ at 20 and 100 K in a 1 T field to further explore their structural and magnetic properties. For these measurements, a spin-polarized neutron beam with a wavelength band λ (0.354–0.942 nm) was produced using a two-mirror v-design transmission supermirror polarizer. The spin state of the incident neutrons was selected to be up (+) or down (−) with a spin flipper. Two samples were measured simultaneously, and the scattered beam was measured at four different values of the incident angle θ to cover an extended *Q* range for each. The spin-dependent neutron reflectivities, R^+^ and R^–^, were then obtained as a function of the wavevector transfer *Q* in the direction normal to the film surface. Only polarized spin-up (R^+^) and spin-down (R^–^) cross sections of the reflectivity were collected since a 1 T field is enough to saturate any parasitic moment in MnTe in the sample plane at the measurement temperatures.

PNR probes the nuclear and magnetic scattering length densities (ρ and ρ_M_, respectively), the latter of which is proportional to the magnitude of the ferromagnetic component of the film magnetization aligned parallel to the applied magnetic field. These components can be extracted through modeling and fitting of the measured reflectivity data, and the ρ and ρ_M_ depth profiles can be reconstructed as a function distance from the film–substrate interface, Z, normal to a sample surface. Data reduction for the experiments was completed using the QuickNXS extraction software, and fitting and uncertainty analysis were performed using the Refl1d software packages.^41,42^ The values of the incident intensities and angle offset were varied independently in fits of the reflectivity data obtained at different values of the incident angle θ.

In typical PNR experiments, there is degeneracy in the models used for fitting, where multiple depth profiles yield similar χ^2^ values. As a result, a unique reconstruction of the sample structure in real space is not possible. This issue requires limiting potential models to those that are consistent with the known physical parameters of the system. During the fitting process we started from the simplest model, nonmagnetic MnTe and a uniform ρ for all layers, and then we produced a series of increasingly complex models to determine the point of under or over parametrization. To further improve the models and uncertainties, fitting was performed on data collected in 1 T at 20 and 100 K simultaneously, which allowed parameters to be constrained. Our models used the assumptions that the sample structure did not change between the two temperatures, except for the surface layer (“SL” in Figure 5)—representative of ice and/or condensate accumulation while cooling. Parameters related to magnetism, such as ρ_M_ and magnetic thickness, were allowed to vary between the two temperatures, as changes in superparamagnetic clusters and/or changes in magnetic anisotropy could produce differences between the two temperatures. A more detailed description of the fitting process and models representing under and over parametrization are included in the “Polarized Neutron Reflectometry” section of the Supporting Information.

## Supplementary Material



## Data Availability

All data of this study is available at https://data.2dccmip.org/8LP0hbN3XhSJ.

## Notes

Table containing growth parameters from Samples A to J; high resolution XRD characterization of Sample A; FWHM of MnTe 0004 and 101̅5 reflections from XRD rocking curves as a function of growth temperature and Te/Mn flux ratio; cross-sectional STEM and EDS mapping of MnTe film on InP; PNR data and spin asymmetry of Te (10 nm)/MnTe (35 nm)/InP taken at 100 K along with theoretical fittings using the Bulk odel and Interfacial Magnetization model; additional explanation on the PNR data fitting process with under parametrized model and over parametrized model included; Electrical transport measurement of Te (10 nm)/MnTe (35 nm)/InP taken from 300 K down to 2 K (PDF)
