# Supplementary material for: Examining the Spin Structure of Altermagnetic Candidate MnTe Grown with Near Ideal Stoichiometry
Source: ACS Nano. 2026 Jul 28;20(31):21625–35. doi: 10.1021/acsnano.6c01072 (PMC13472319; doi:10.1021/acsnano.6c01072)
Supplement: Supplementary file 1 [file nn6c01072_si_001.pdf]

Supporting Information

Examining the Spin Structure of Altermagnetic Candidate MnTe Grown with Near Ideal Stoichiometry

Qihua Zhang[1*], Christopher J. Jensen[2], Alexander J. Grutter[2], Sandra Santhosh[3], William D. Ratcliff[2,4,5], Julie A. Borchers[2], Thomas W. Heitmann[6], Narendirakumar Narayanan[6], Timothy R. Charlton[7], Mingyu Yu[8], Ke Wang[9], Wesley Auker[9], Saurav Islam[1,3,9], Nitin Samarth[1,3,9,10,11], Stephanie Law[1,3,9,10,12]*

[1]Two Dimensional Crystal Consortium Materials Innovation Platform, The Pennsylvania State University, University Park, Pennsylvania 16802, USA

[2]NIST Center for Neutron Research, NCNR, National Institute of Standards and Technology, Gaithersburg, Maryland 20899, USA

[3]Department of Physics, The Pennsylvania State University, University Park, Pennsylvania 16802, USA

[4]Department of Physics, University of Maryland, College Park, Maryland 20742, USA

[5]Department of Materials Science and Engineering, University of Maryland, College Park, Maryland 20742, USA

[6]University of Missouri Research Reactor (MURR), University of Missouri, Columbia, Missouri 65211, USA

[7]Neutron Scattering Division, Oak Ridge National Laboratory, Oak Ridge, Tennessee 37831, USA

[8]Department of Materials Science and Engineering, University of Delaware, 201 Dupont Hall, 127 The Green, Newark, Delaware 19716, USA

[9]Materials Research Institute, The Pennsylvania State University, University Park, Pennsylvania 16802 USA

[10]Department of Materials Science and Engineering, The Pennsylvania State University, University Park, Pennsylvania 16802 USA

[11]Argonne National Lab, Chicago, Illinois 60439 USA

[12]Institute of Energy and the Environment, The Pennsylvania State University, University Park, Pennsylvania 16802 USA

CORRESPONDING AUTHORS

Qihua Zhang – **qzz5173@psu.edu**

Stephanie Law – **sal6149@psu.edu**

Table S1. Growth parameters (Mn flux, Te flux, Growth Temperature, Te/Mn FR) of the MnTe layer in Samples A-L. The RMS roughness of the 2 μm × 2 μm AFM images are also included.

Sample No.	Mn Flux (cm^{-2}/s)	Te Flux (cm^{-2}/s)	Growth Temperature (°C)	Te/Mn FR	RMS roughness (nm)	MnTe (0004) 2θ position
A	7.6×10^{13}	2.3×10^{14}	200	3	0.8	54.70°
B	7.6×10^{13}	2.3×10^{14}	250	3	0.9	54.76°
C	7.6×10^{13}	2.3×10^{14}	300	3	1.0	54.80°
D	7.6×10^{13}	2.3×10^{14}	340	3	1.4	54.80°
E	7.6×10^{13}	2.3×10^{14}	400	3	1.9	54.81°
F	7.6×10^{13}	2.3×10^{14}	500	3	3	54.86°
G	7.6×10^{13}	7.6×10^{13}	340	1	0.8	54.87°
H	7.6×10^{13}	1.5×10^{14}	340	2	2.0	54.83°
J	7.6×10^{13}	3.1×10^{14}	340	4	1.2	54.79°

1. Structural Characterizations of MnTe films

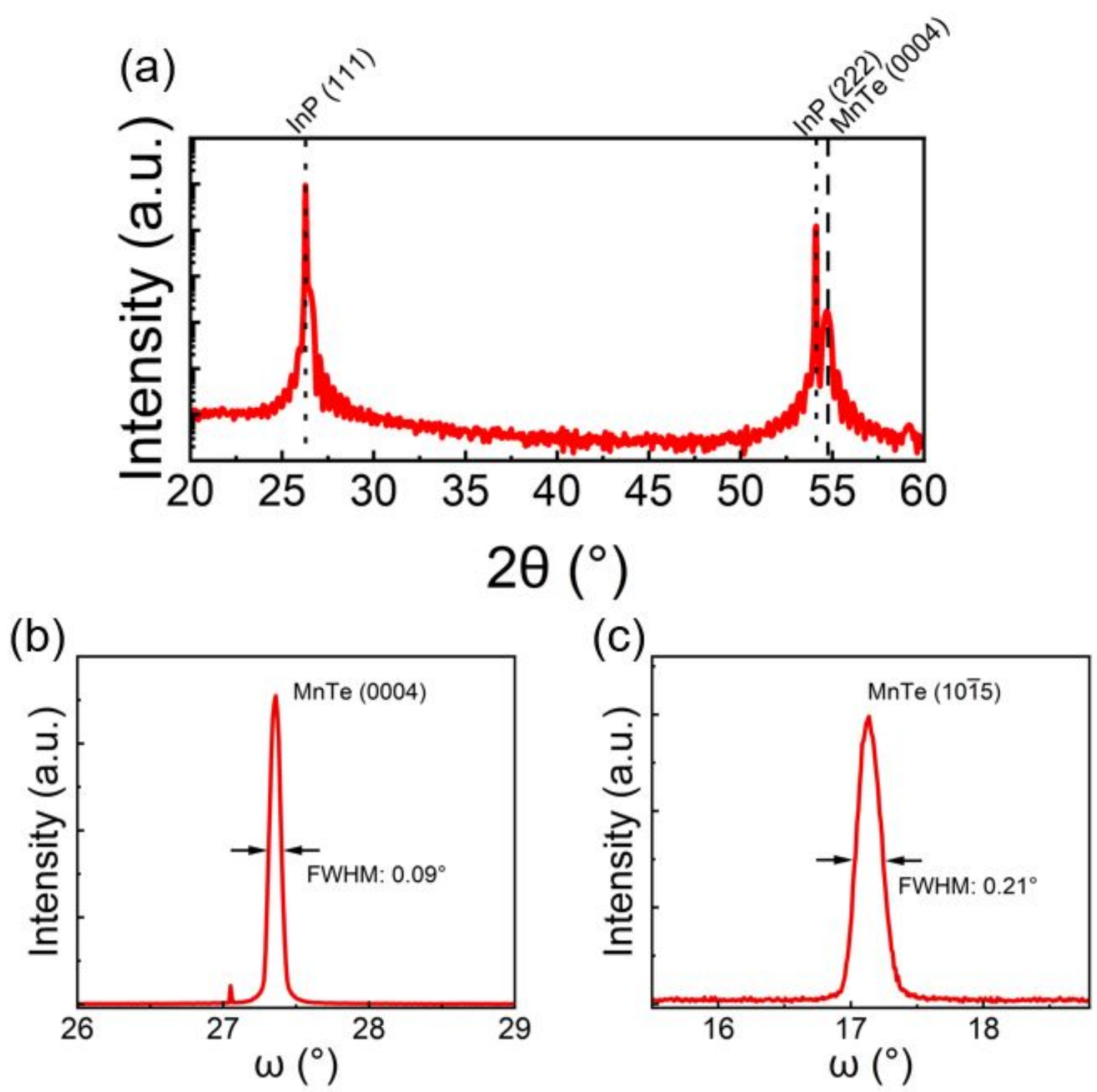

Figure S1. High resolution XRD characterization of Sample A. (a) XRD 2θ-ω coupled scan. The InP diffraction peaks are marked by dotted lines; MnTe diffraction peaks are marked by dashed lines. (b) ω-scan of the MnTe 0004 reflection. (c) ω-scan of the MnTe $10\bar{1}5$ reflection.

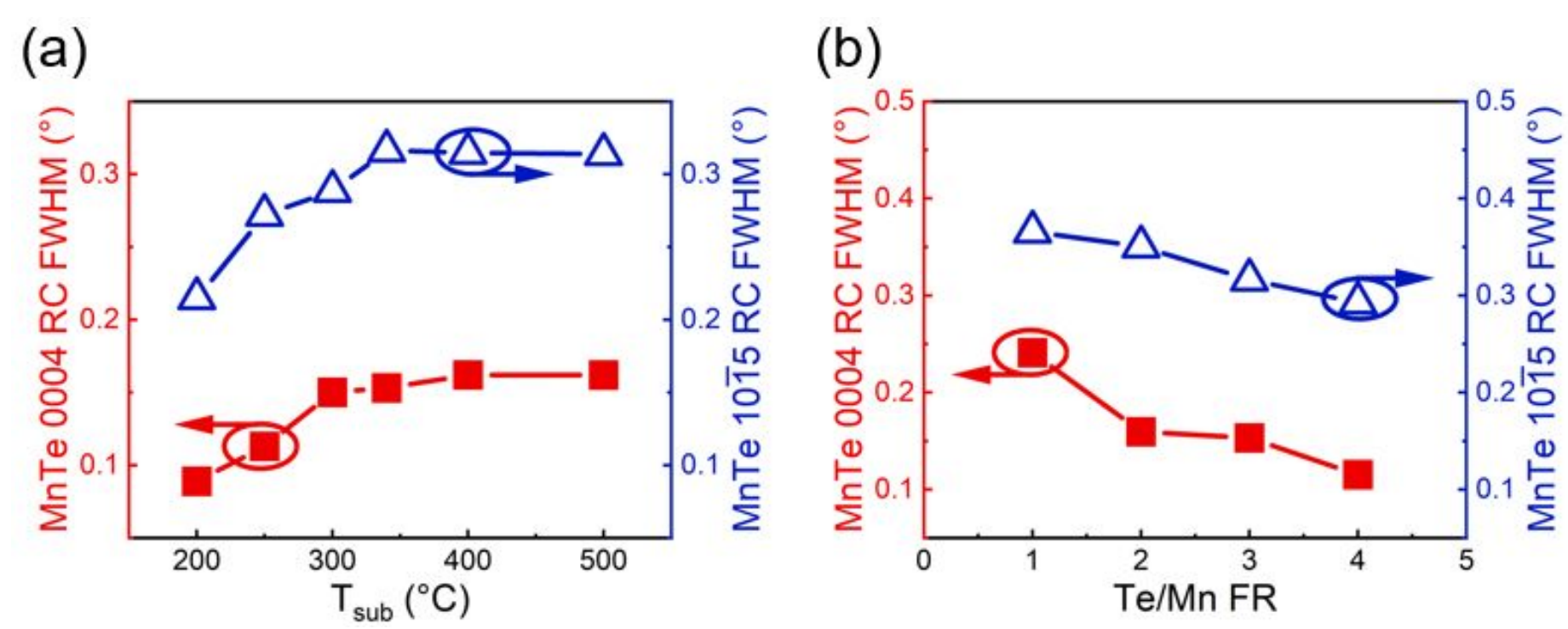

Figure S2. (a) FWHM of the MnTe 0004 (in red solid squares) and $10\bar{1}5$ (in blue open triangles) reflections from XRD rocking curves as a function of growth temperature. (b) FWHM of the MnTe 0004 (in red solid squares) and $10\bar{1}5$ (in blue open triangles) reflections from XRD rocking curves as a function of Te/Mn flux ratio.

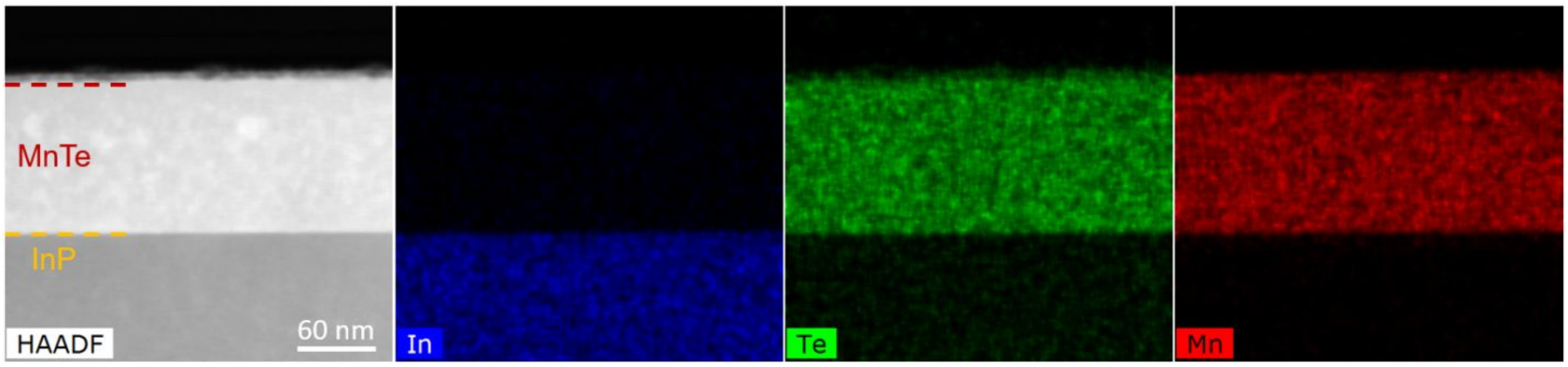

Figure S3. Cross-sectional HAADF-STEM image (left) and EDS mapping of In, Te, and Mn. The dashed lines in the HAADF image separate the MnTe film and InP substrate.

2. Polarized Neutron Reflectometry (PNR)

100 K –Reflectivity and Spin Asymmetry

The fits for the reflectivity measured at 100 K of the Te (10 nm)/ MnTe (35 nm)/ InP sample are shown in Fig. S4 (bulk model) and S6 (interfacial magnetization model). These fits, and the fits of the 20 K reflectivity data (Fig. 6), were generated from the nuclear and magnetic scattering length densities (ρ and ρ_M) shown in Fig. 5 of the main text. Fig. S5 (bulk model) and S7 (interfacial magnetization model) are the calculated spin asymmetry [$(R^+ - R^-) / (R^+ + R^-)$] for the data and fits.

Bulk Model

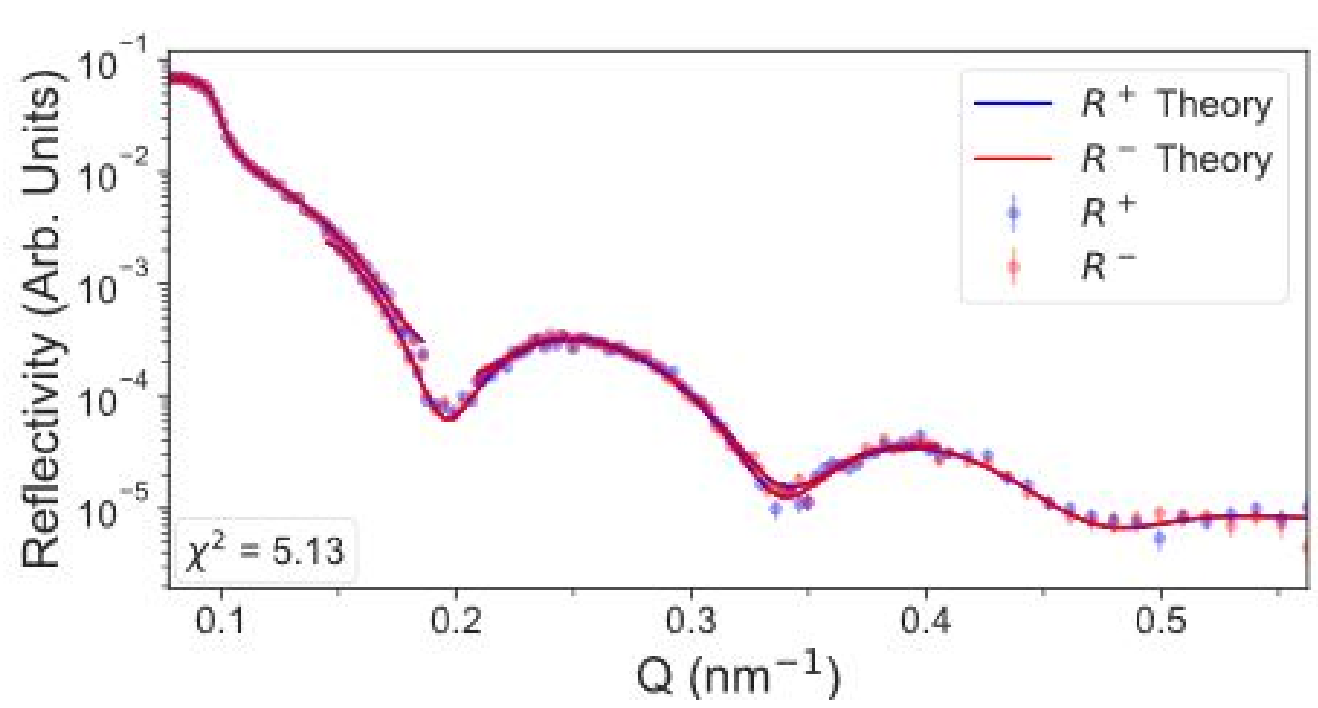

Figure S4. PNR data (points) and theoretical fits (solid lines) calculated using the bulk layer model for measurements at 100 K on Te (10 nm)/MnTe (35 nm)/InP.

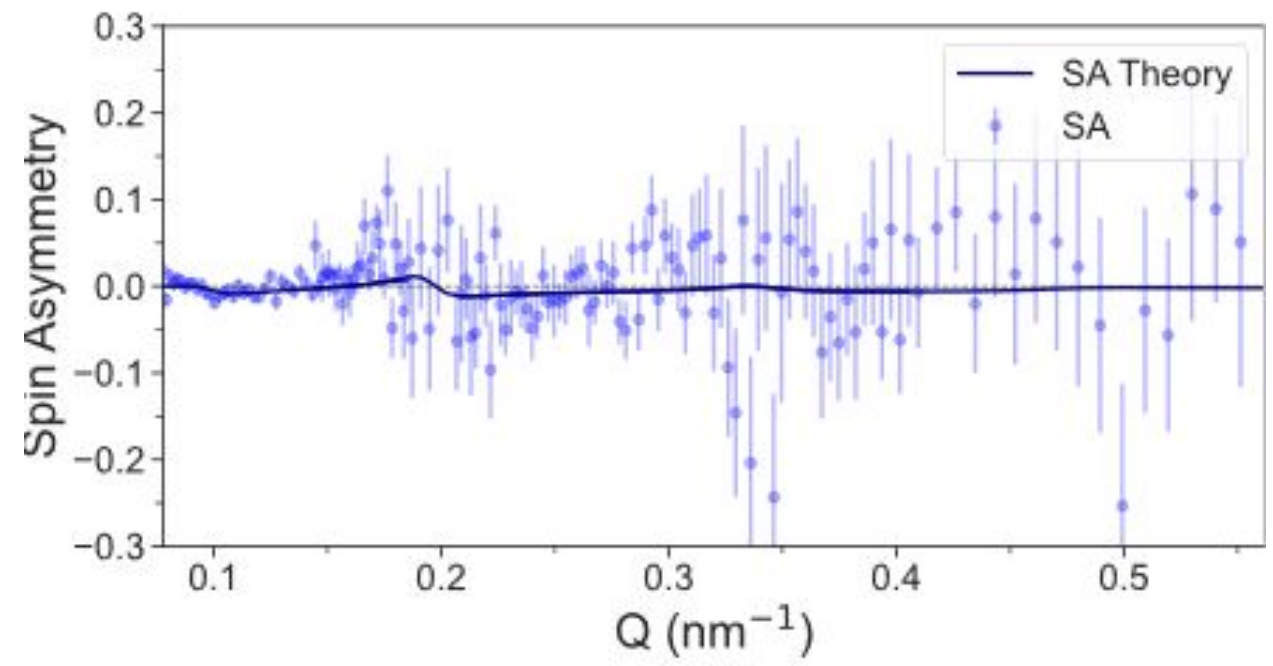

Figure S5. Spin asymmetry calculated from the data (points) and theoretical fits (solid lines) using the bulk model for the PNR measurements taken at 100 K on Te (10 nm)/MnTe (35 nm)/InP films. Spin asymmetry is defined as $(R^+ - R^-) / (R^+ + R^-)$.

Interfacial Magnetization Model

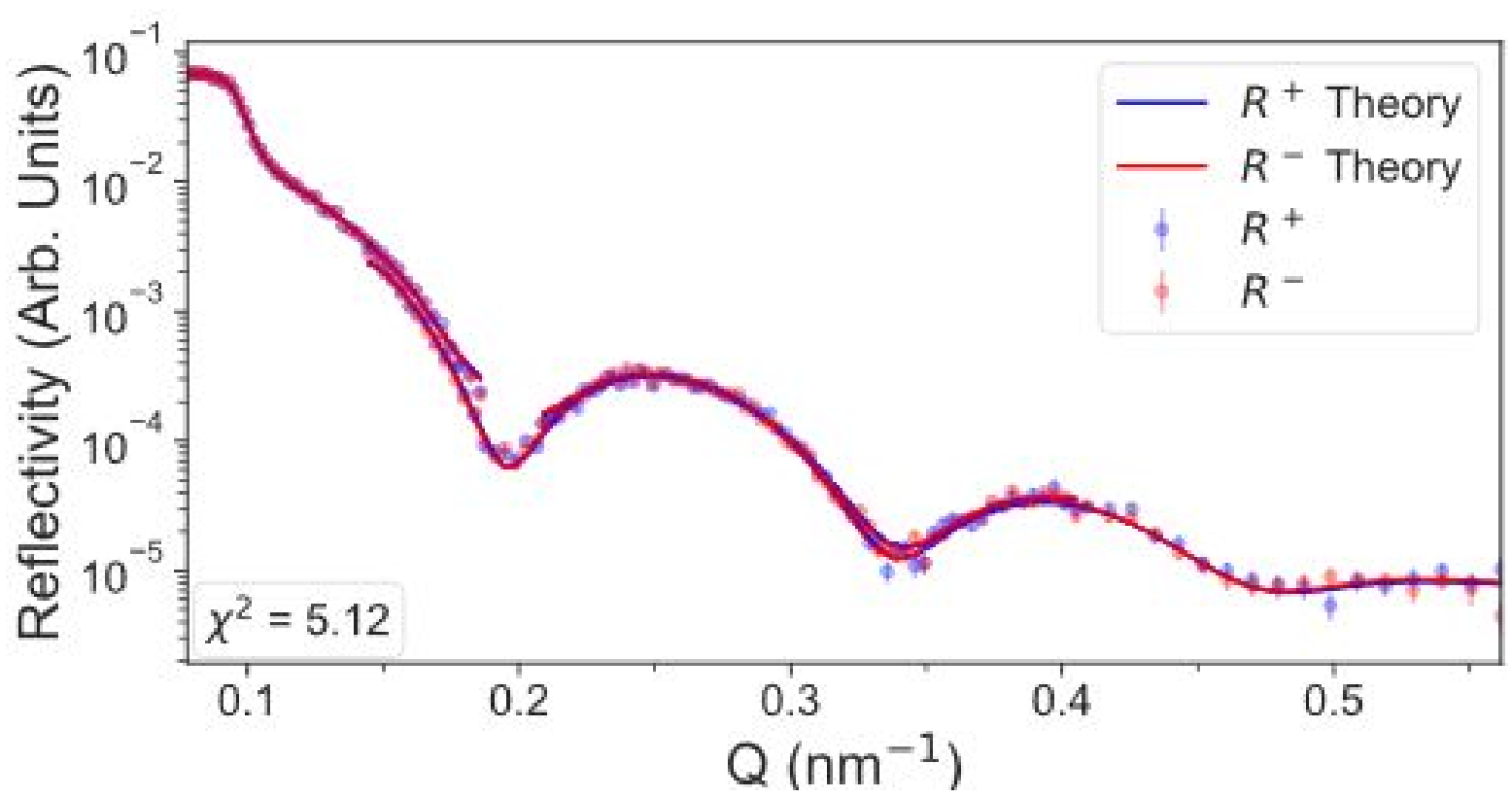

Figure S6. PNR data (points) and theoretical fits (solid lines) calculated using the interfacial magnetization model for measurements at 100 K on Te (10 nm)/MnTe (35 nm)/InP films).

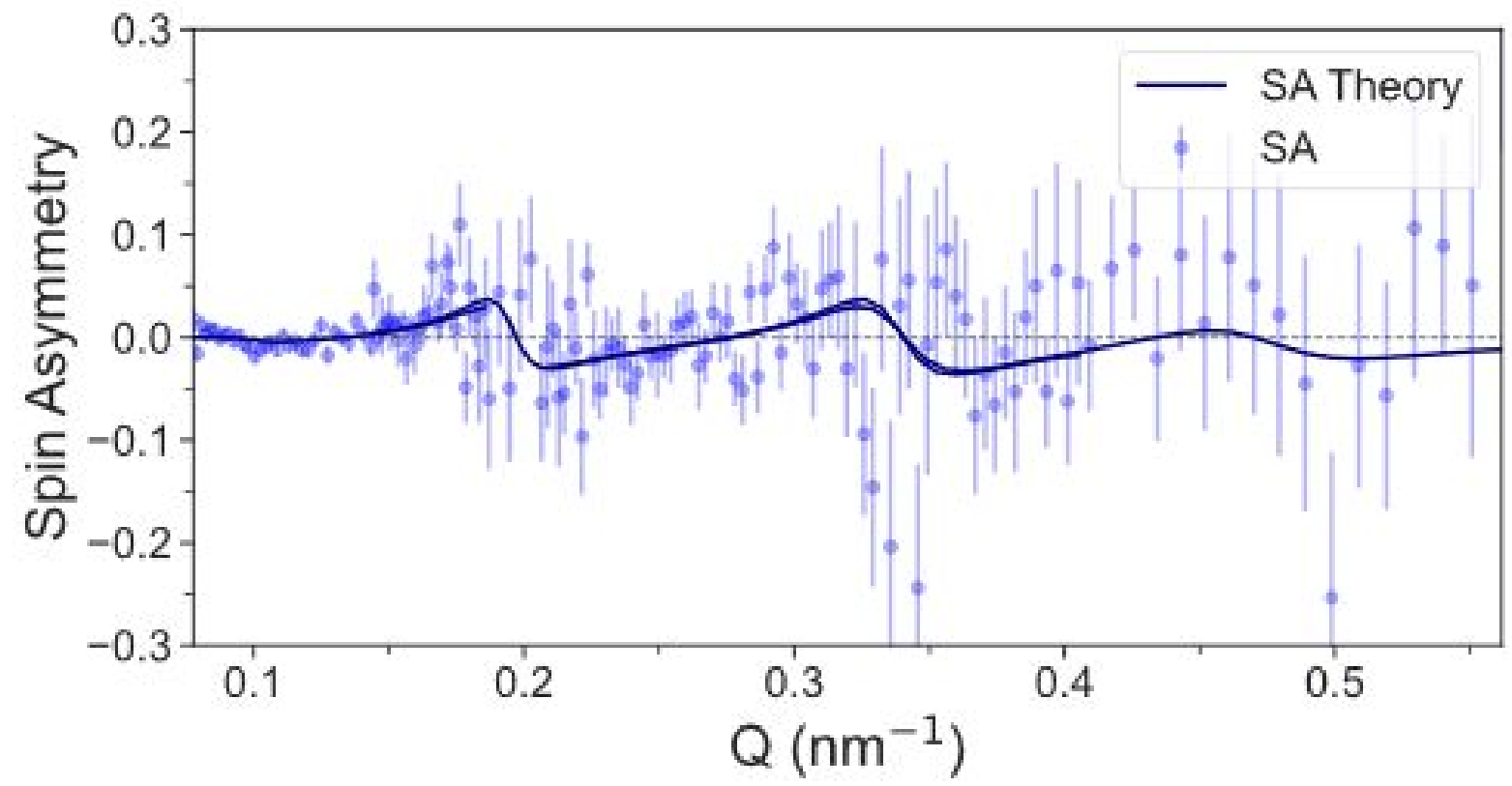

Figure S7. Spin asymmetry calculated from the data (points) and theoretical fits (solid lines) using the interfacial magnetization model for the PNR measurements taken at 100 K on a Te (10 nm)/MnTe (35 nm)/InP film. Spin asymmetry is defined as (R^+ - R^-) / (R^+ + R^-).

Further Explanation on Fitting Process

Polarized neutron reflectivity data were fit using a series of models, starting from a simple structure with uniform ρ for each layer in the structure and no ρ_M (i.e., no magnetization allowed to be fit in MnTe). From this, a series of more complex models were considered to identify the best fit model(s) – as shown in the main text Fig. 5 – and those that were believed to be under or over parameterized. To determine the quality of each fit, the goodness of fit (χ^2) was considered, but also the qualitative match between the shape of the calculated theoretical reflectivities and the spin asymmetries and data. The latter is especially relevant for this study, as extracting the magnitude and distribution of the magnetization is critical for understanding altermagnetic characteristics of MnTe. Below we discuss the quality of the fits in the context of two models that were under-parameterized and over-parameterized, referred to as "non-magnetized MnTe" and "interfacial MnTe layer", respectively.

Under Parameterized Model – Non-Magnetized MnTe

The best-fit ρ and ρ_M profiles from the non-magnetized MnTe model are shown in Fig. S8 for the 100 K and 20 K measurements of the Te (10 nm)/ MnTe (35 nm)/ InP sample. A uniform ρ was used for each layer in the sample, and the interfacial widths of each layer were fit, except for that of TeO_x, which was linked to the interfacial width of the Te layer. By way of definition, the interfacial width is the full width of the error function used to describe the layer interfaces assuming Gaussian roughness. The origin of this interfacial mixing could be conformal roughness, thickness variations and/or chemical interdiffusion averaged across the entire 1 cm^2 sample plane. The width of the InP/MnTe interface obtained from all fits described here was 0.5 ± 0.3 nm, and the width of the MnTe/Te interface was 2.1 ± 0.4 nm. These values confirm that the MnTe film is structurally uniform through the majority of its depth.

The fits of the 100 K and 20 K data were conducted simultaneously, with all layers' structural properties assumed to be equal between 100 K and 20 K, except for the surface layer ("SL" in Fig. S8). As described in the main text, this surface layer is likely a result of ice or condensate accumulation on the surface of the film while cooling, and the increased thickness at 20 K is consistent with this explanation. The MnTe was fit with a $\rho = 0.439 \times 10^{-4}$ nm^{-2}, which is higher than the expected value for bulk but about the same as the bulk and interfacial magnetization models in the main text. The ρ_M for all layers were forced to be zero, which is representative of a film with a MnTe layer that is devoid of any magnetization.

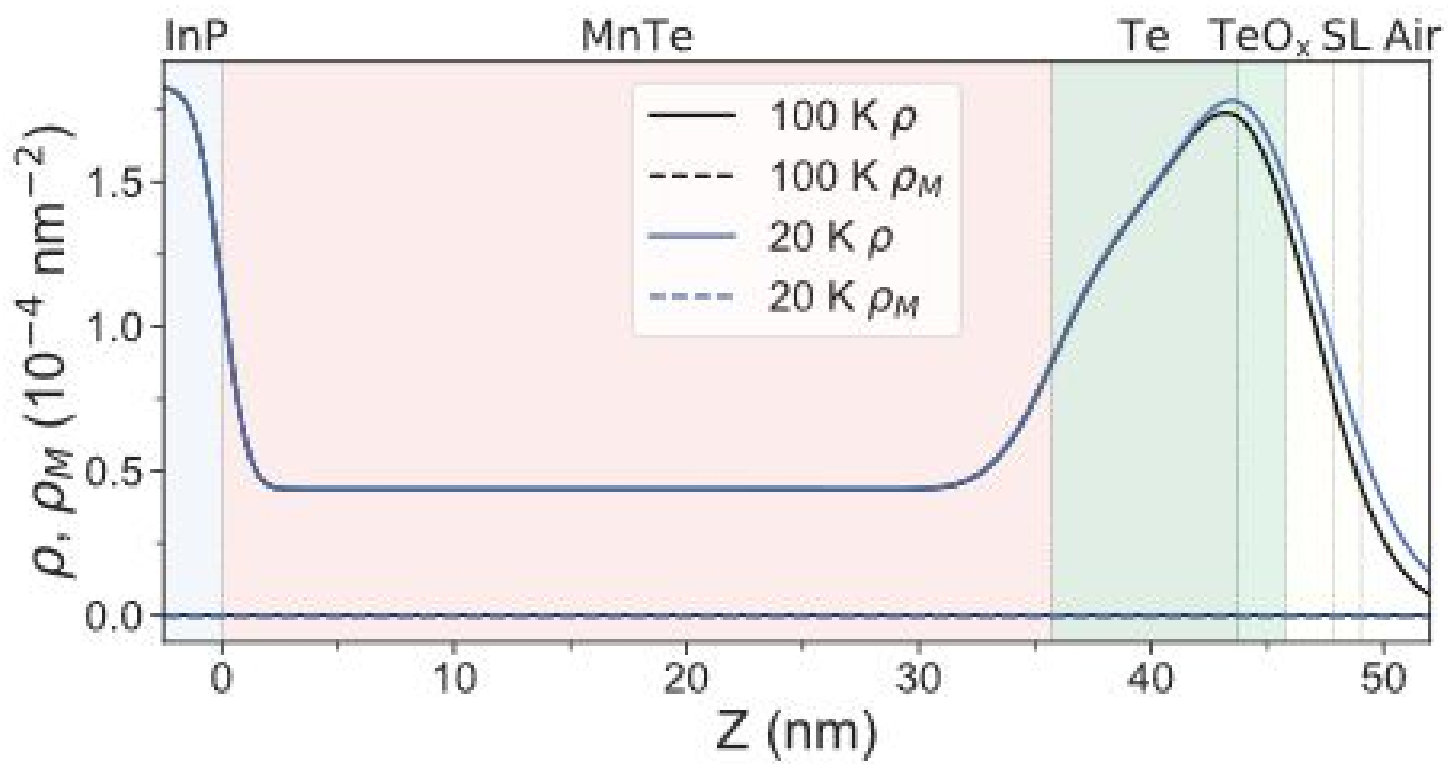

Figure S8. PNR ρ and ρ_M profiles calculated from fits of data collected for the Te (10 nm)/MnTe (35 nm)/InP film at 100 K (black) and 20 K (blue) using the non-magnetized MnTe model. Solid lines represent ρ and the dashed lines (hidden by origin) represent ρ_M. Background colors with corresponding labels above the plot were used to highlight the expected layer positions along the depth (Z) of the sample. Note that "SL" represents a likely surface layer of ice/condensation that formed during sample cooling.

The 20 K (Fig. S9(a)) and 100 K (Fig. S9(c)) reflectivity are both fit well by this model, but with a slightly higher value of χ^2 compared to the best fits included in the main text (Fig. 6).When spin asymmetry is calculated (Fig. S9(b) and S9(d)), the theoretical curve is a flat line at zero since there is no splitting in the fitted R^+ and R^- reflectivities. This clearly does not match the fluctuations in the spin asymmetry observed in the data and contributes to the slight increase in χ^2, so this model was considered under parameterized. Additionally, it justifies that a magnetic layer is needed in the model to produce the appropriate splitting measured between the R^+ and R^-.

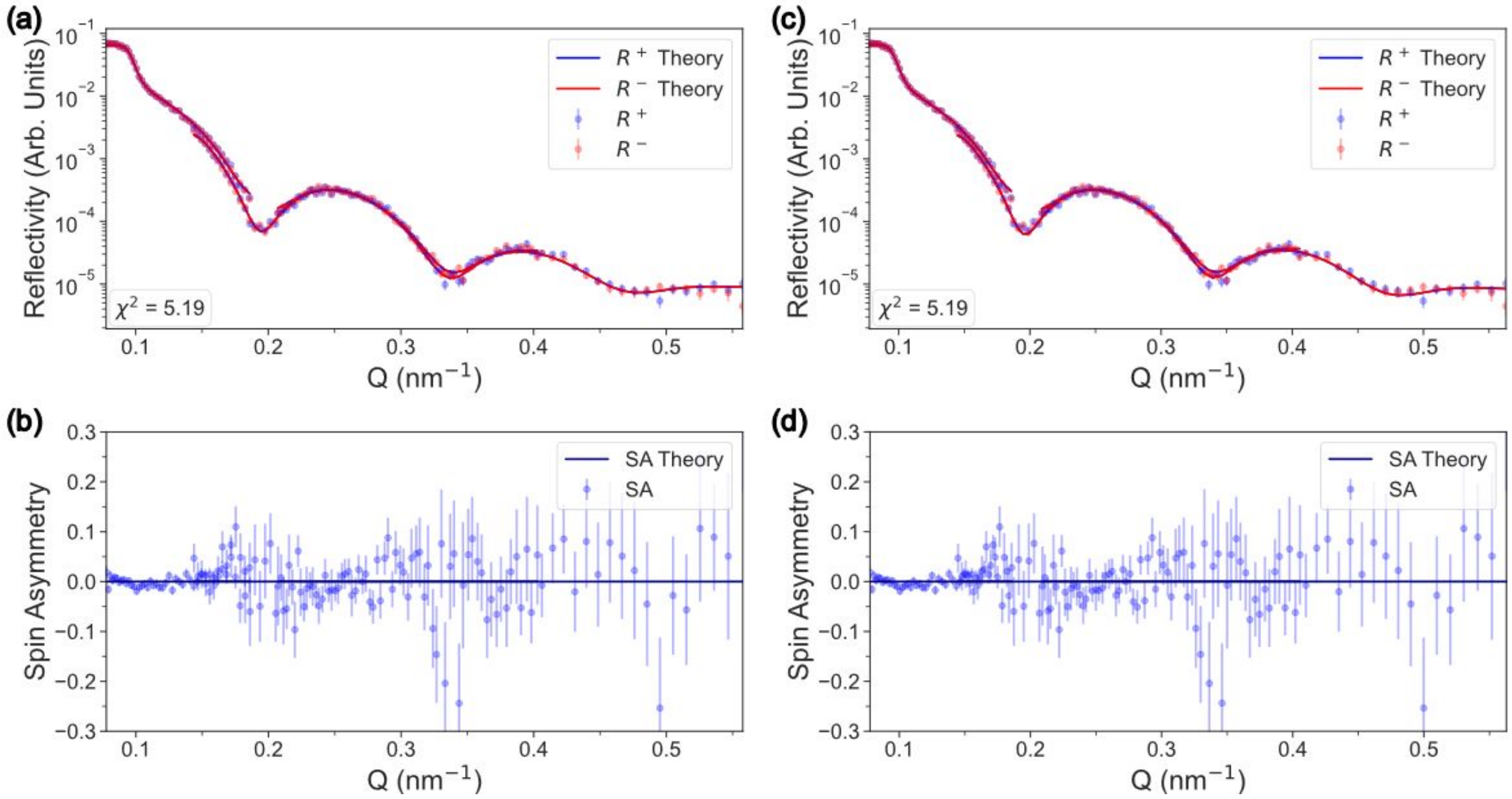

Figure S9. PNR data (points) and theoretical fits (solid lines) calculated using the non-magnetized MnTe model for measurements at (a) 20 K and (c) 100 K on Te (10 nm)/MnTe (35 nm)/InP films). Spin asymmetry was calculated from these data (points) and theoretical fits (solid lines) for the (b) 20 K and (d) 100 K on a Te (10 nm)/MnTe (35 nm)/InP film. Spin asymmetry is defined as $(R^+ - R^-) / (R^+ + R^-)$.

Over Parameterized Model – Interfacial MnTe Layer

The interfacial MnTe layer model's calculated ρ and ρ_M profiles are shown in Fig. S10 for the 100 K and 20 K measurements of the Te (10 nm)/ MnTe (35 nm)/ InP sample. This model introduced a thin $MnTe_{int}$ layer at the MnTe/InP interface that may be fit with different ρ and ρ_M values than those of the MnTe layer. By introducing $MnTe_{int}$, this model is very similar to the interfacial magnetization model chosen as one of the best fits (Fig. 5(c)) but differs since ρ was allowed to vary in this interface region. During the fitting process, the fit values for each layer were constrained to be the same between the 100 K and 20 K (assuming no large structural changes), except for the values for ρ_M in MnTe and the structural parameters for the surface layer ("SL" Fig.

S10). The increase in the surface layer thickness at 20 K is again consistent with the ice or condensate accumulation during the cooling process. MnTe in this model resulted in ρ = 0.443 × 10^{-4} nm^{-2}, which is comparable to that obtained from the other models. The $MnTe_{int}$ ρ = 0.119 × 10^{-4} nm^{-2} with a thickness of 1.0 nm between the InP and MnTe layers. This reduction in ρ could correspond to a transition layer being formed during growth with lower density or a higher percentage of Mn. Magnetization in this model is confined to $MnTe_{int}$, with $\rho_M = 0.128 \times 10^{-4}$ nm^{-2} at 100 K and $\rho_M = 0.095 \times 10^{-4}$ nm^{-2} at 20 K, corresponding to M_{fit} of 45 kA/m and 33 kA/m, respectively. These values are comparable to the magnetization obtained using the interfacial magnetization model (Table 1) and are consistent with similar findings of ferromagnetism being present in Mn-rich MnTe films.[1]

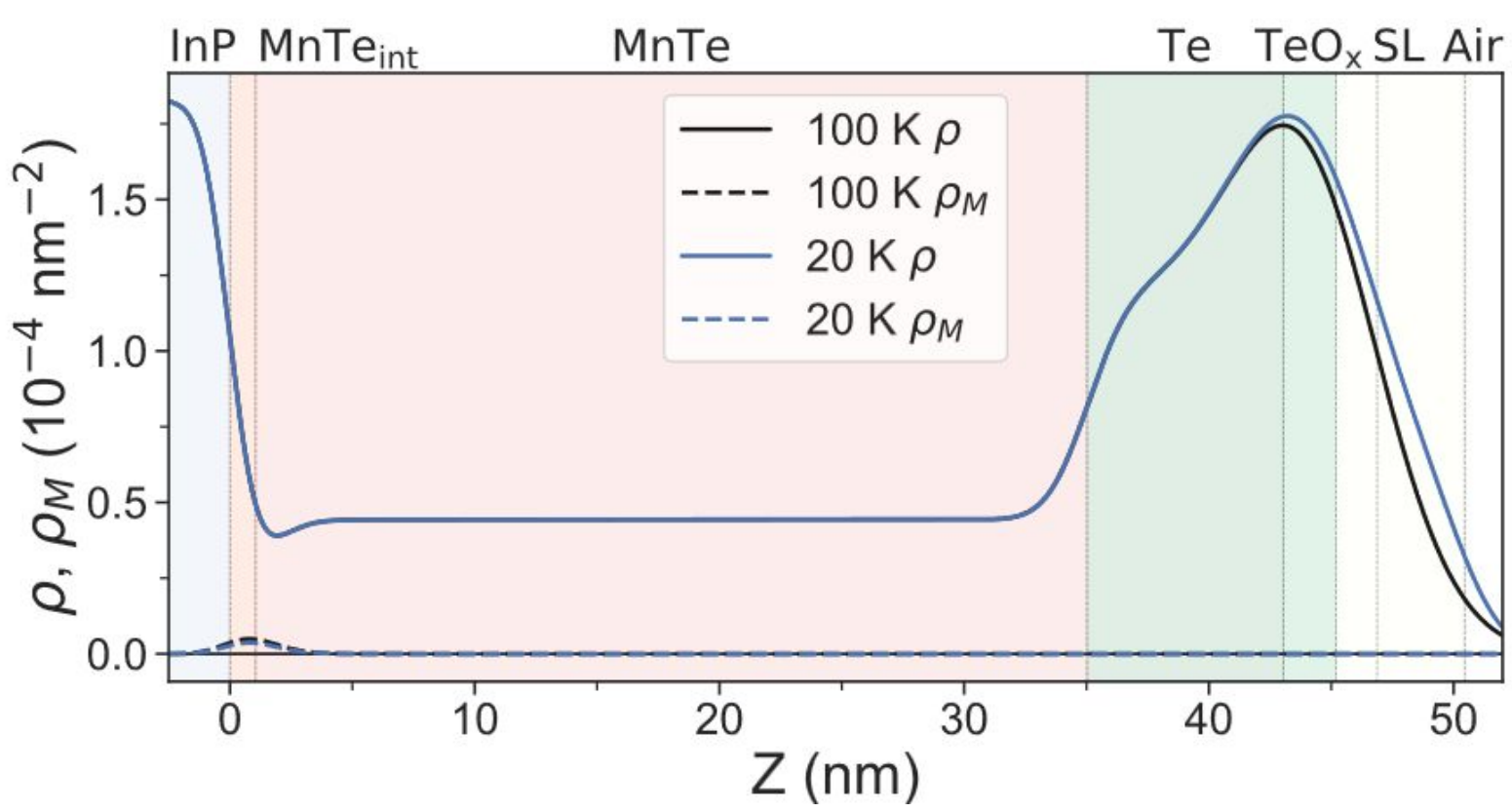

Figure S10. PNR ρ and ρ_M profiles calculated from fits of data collected for the Te (10 nm)/MnTe (35 nm)/InP film at 100 K (black) and 20 K (blue) using the interfacial MnTe layer model. Solid lines represent ρ and the dashed lines represent ρ_M. Background colors with corresponding labels above the plot were used to highlight the expected layer positions along the depth (Z) of the sample. Note that "SL" represents a likely surface layer of ice/condensation that formed during sample cooling.

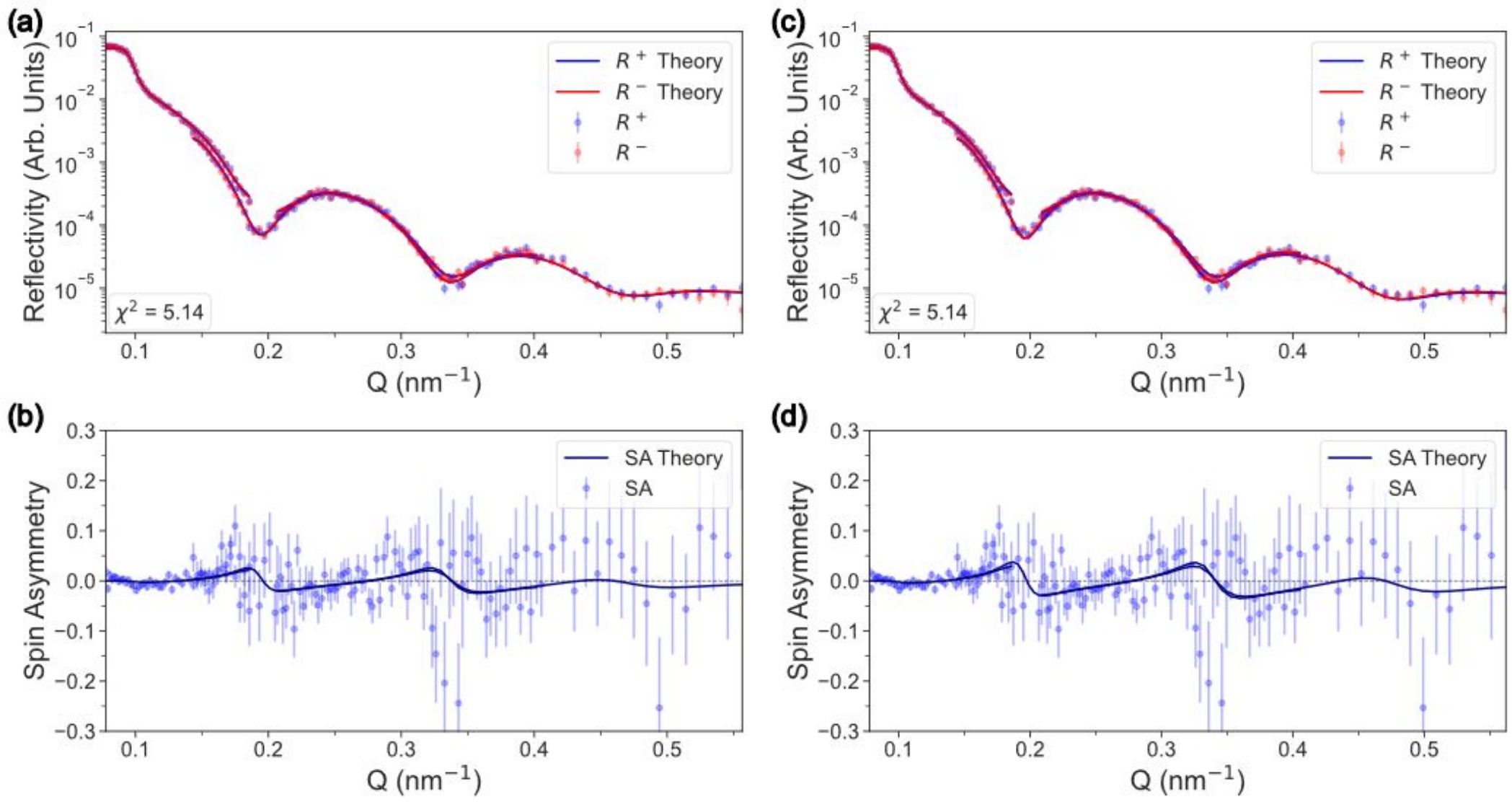

Figure S11. PNR data (points) and theoretical fits (solid lines) calculated using the interfacial MnTe layer model for measurements at (a) 20 K and (c) 100 K on Te (10 nm)/MnTe (35 nm)/InP films). Spin asymmetry was calculated from these data (points) and theoretical fits (solid lines) for the (b) 20 K and (d) 100 K on a Te (10 nm)/MnTe (35 nm)/InP film. Spin asymmetry is defined as $(R^+ - R^-) / (R^+ + R^-)$.

When compared to the interfacial magnetization model (Fig. 5(c), 5(d) and Fig. S6 and S7), the reflectivity and spin asymmetry in Fig. S11 are very similar, and χ^2 is almost identical. Because of this, it is hard to justify this model over the interfacial magnetization model, and the addition of ρ variation in the $MnTe_{int}$ layer is not critical to the overall fit. We consider this model to be over-parameterized though we cannot rule out the possibility of a thin interfacial, transition layer with reduced structural density and isolated ferromagnetism.

3. Electrical transport measurements of MnTe thin films

The electrical properties of Te capped MnTe films (35 nm) on Fe-doped semi-inuslating InP (111)A substrate were measured with a Quantum Design Dynacool Physical Property Measurement System using an excitation current of 10 μA. Hall bar geometry was employed for

device characterization, with an area of 4 × 2 mm^2. Figure S12(a) shows the longitudinal resisntance (R_{xx}) as a function of temperature from 300 K down to 2 K. A typical metallic behavior is observed, with an estimated residual resitivity ratio of ~4, which is higher than the MnTe films from previous reports (e.g. ~3.3 in Ref. [1]) and other altermagnetic candidates (e.g. ~2 in CrSb in Ref. [2]), denoting the improved film quality in this study. A slight upturn in R_{xx} at temperatures below 15 K can also be observed, possibly due to electron-electron interacions. The measured carrier concentration in the MnTe film is further shown in Figure S12(b). The 35 nm MnTe film exhibits a p-type conductivity with hole concentrations in the range of 10^{19} cm^{-3}, which agrees well with previous reports. The reason for obtaining a p-type film could be due to a number of defects such as Mn vacancies and Mn-Te antistite defects.

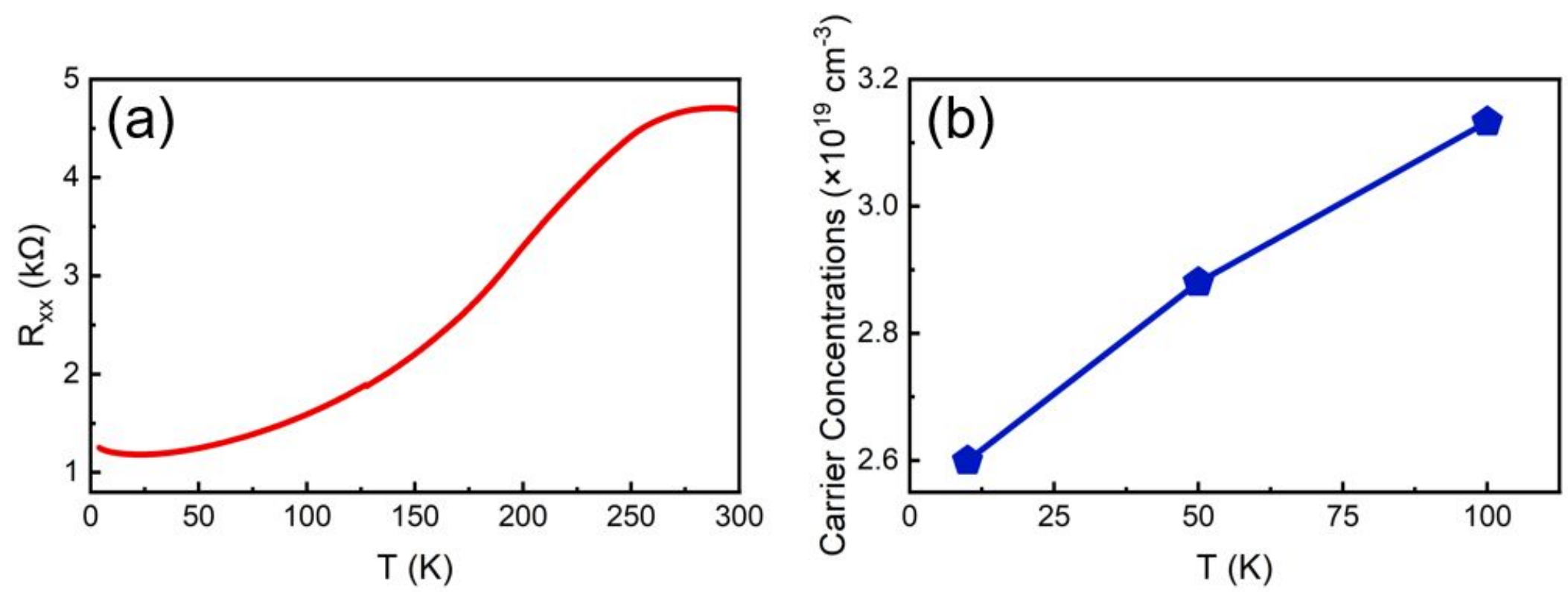

Figure S12. Electrical transport measurements on a 35 nm MnTe film with Te capping. (a) Longitudinal resistance (R_{xx}) as a function of temperature (T). (b) Carrier concentration as a function of temperature (T).

References

(1) Chilcote, M.; Mazza, A. R.; Lu, Q.; Gray, I.; Tian, Q.; Deng, Q.; Moseley, D.; Chen, A.-H.; Lapano, J.; Gardner, J. S.; Eres, G.; Ward, T. Z.; Feng, E.; Cao, H.; Lauter, V.; McGuire, M. A.; Hermann, R.; Parker, D.; Han, M.-G.; Kayani, A.; Rimal, G.; Wu, L.; Charlton, T. R.; Moore, R. G.; Brahlek, M. Stoichiometry-Induced Ferromagnetism in Altermagnetic Candidate MnTe. *Adv. Funct. Mater.* **2024**, *34* (46), 2405829.

(2) Santhosh, S.; Corbae, P.; Yánez-Parreño, W. J.; Ghosh, S.; Jensen, C. J.; Fedorov, A. V.; Hashimoto, M.; Lu, D.; Borchers, J. A.; Grutter, A. J.; Charlton, T. R.; Islam, S.; Golovanova, D.; Zhao, Y.; Tauraso, A.; Richardella, A.; Yan, B.; Mkhoyan, K. A.; Palmstrøm, C. J.; Ou, Y.; Samarth, N. Altermagnetic Band Splitting in 10 nm Epitaxial CrSb Thin Films. *Adv. Mater.* **2025**, *37* (47), e08977.
